# Evolutionary design optimization of traffic signals applied to Quito city

**DOI:** 10.1371/journal.pone.0188757

**Published:** 2017-12-13

**Authors:** Rolando Armas, Hernán Aguirre, Fabio Daolio, Kiyoshi Tanaka

**Affiliations:** 1 Faculty of Engineering, Shinshu University. 4-17-1 Wakasato, Nagano 380-8553, Japan; 2 Computing Science and Mathematics, University of Stirling, Stirling, Scotland; Southwest University, CHINA

## Abstract

This work applies evolutionary computation and machine learning methods to study the transportation system of Quito from a design optimization perspective. It couples an evolutionary algorithm with a microscopic transport simulator and uses the outcome of the optimization process to deepen our understanding of the problem and gain knowledge about the system. The work focuses on the optimization of a large number of traffic lights deployed on a wide area of the city and studies their impact on travel time, emissions and fuel consumption. An evolutionary algorithm with specialized mutation operators is proposed to search effectively in large decision spaces, evolving small populations for a short number of generations. The effects of the operators combined with a varying mutation schedule are studied, and an analysis of the parameters of the algorithm is also included. In addition, hierarchical clustering is performed on the best solutions found in several runs of the algorithm. An analysis of signal clusters and their geolocation, estimation of fuel consumption, spatial analysis of emissions, and an analysis of signal coordination provide an overall picture of the systemic effects of the optimization process.

## Introduction

Transportation and mobility systems have become large-scale and complex in many cities, with broad social impacts and strong implications for the economy and the environment. Urban population is overgrowing around the globe [1] bringing an increase in transportation and mobility demand that improperly satisfied often causes congestion of the system. This adds substantial costs due to delays, increases gas emissions, the risk of accidents and affects health. Thus, efforts to continuously redesign the transportation system and make it sustainable to guarantee the mobility of larger populations and the accessibility of cities are required. These efforts include the increase of infrastructure capacity, land-use planning, improving public transportation, and the incorporation of soft-computing methods to implement Intelligent Transportation Systems (ITS).

We focus on computational intelligence, machine learning (ML) and data mining methods coupled to a transport simulator to develop a design optimization framework for the transportation system of Quito (Ecuador’s capital), using the outcome of the optimization process for problem understanding and to gain knowledge to suggest innovations to the real system.

Quito’s urban population growth has put enormous strains on its transportation system. There is the need to model the system, better understand the problems associated with transportation and mobility, find sustainable solutions to solve them and improve life in the city.

In this work, our objective is to minimize travel time optimizing settings of 70 signalized intersections located in the business centre of Quito aiming to reduce traffic congestion, fuel consumption and emissions. We model activity-based mobility plans of inhabitants with private cars and simulate them on Quito’s transport network using the microscopic Multi Agent Transport Simulator (MatSim) [2]. We concentrate on the optimization of a large number of traffic lights deployed on a wide area of the city and study their impact on travel time, emissions and fuel consumption. A proper setting of traffic signals can help to alleviate the traffic congestion with better use of the current infrastructure. Moreover, it is a key component to study other important problems related to mobility in order to improve the sustainability of the transport system.

We use evolutionary algorithms (EA) to explore a significant number of alternative signal settings under various scenarios of mobility. Activity based micro-simulation allows us to model the mobility demand of each person in the scenario and facilitates a detailed analysis of the traffic in the city. However, it is computationally expensive. This imposes limits on the number of fitness evaluations, population size, number of generations, and on the number of times the stochastic evolutionary algorithm can be run. In addition, the search space is vast due to the number of signals considered and the several parameters that define a traffic signal. Hence, an important aim of this work is to develop an evolutionary algorithm to search effectively in large decision spaces under a small budget of iterations (generations), performing a reliable short-term evolution to find high-quality solutions. To achieve this, we design a set of specialized mutation operators to search for clusters of coordinated signals with similar cycle length by propagating cycle length between neighboring signals and setting offsets based on the distance between them. Due to the topography of the city and its mobility patterns, it is important to define neighborhoods as two-dimensional and optimize in both directions of traffic flow (north-south-north and east-west-east). Also, we introduce varying mutation rates with high selection pressure to accelerate convergence of the algorithm. The appropriate combination of operators, selection, and varying mutation rates allows to search effectively even with small populations. We use machine learning for parameter analysis of the evolutionary algorithm to compare our settings with ones suggested by ML methods.

To gain knowledge, we use data mining methods to perform hierarchical clustering in decision space of the best solutions found by the evolutionary algorithm. We include the analysis of signal clusters and their geolocation, estimation of fuel consumption, spatial analysis of emissions, and analysis of signal coordination. This gives an overall picture of the systemic effects of the optimization process.

We verify the effectiveness of the developed algorithm for short-term evolution using a small population. We also show that the design optimization approach is a useful tool to advance the understanding of the transport and mobility problems of Quito city, essential for decision making.

### Related work

Several techniques have been proposed for signal timing optimization. These range from statistical based methods in the 60’s [3, 4] to computational intelligence based methods in the last years [5] oriented to implement intelligent transportation systems. In the following, we review the literature where meta-heuristics and biologically inspired algorithms have been combined with traffic simulators for optimization. The review is brief and tries to give a broad perspective, but it does not mean that is comprehensive.

The level of traffic simulation has been either macroscopic [6–9], mesoscopic [10], or microscopic [11–14]. Some works model toy or virtual scenarios mostly for proofs of concept to verify the hypothesis about mobility and transport issues, and to test optimization methods [6, 10, 15–20]. Other works model real world problems [7, 8, 11, 12] and have mainly focused on small areas of interest with relatively few signalized intersections. For example, seven signalized intersections are optimized in [11] and nine in [7]. In these studies, usually, the phases of the signals have been modeled in detail, including several phases for some of the signals. However, typically one common cycle has been considered for all signals and some studies do not consider offsets between them. In [11] different cycle lengths are implicitly modeled, but no offsets are considered. Examples of real-world problems with a relatively large number of signalized intersections are [13, 14], [8] and [21]. In [14] twenty to forty signal controls were considered in an area of 0.42 Km^2^, signals were modeled to allow different cycle lengths with two or more phases, but offsets were not explicitly considered. In [8] seventy-five signalized intersections with two or more phases and offsets between signals were considered in an area of 30 Km^2^. However, a common cycle was used for all signals. In [21] seventy consecutive signalized intersections located on a 13-mile corridor were optimized. The modeling of the signals is detailed, including 12 NEMA movements and right turns, basic and Transit Signal Priority (TSP) timing parameters, with more than two phases in some intersections. However, it uses a binary coding representation for integers applying one-point crossover and bit flipping mutation. Unfortunatelly, with this representation the magnitude of change depends more on the position of the bit being mutated than on the mutation probability. Thus, tuning of the algorithm and robustness to parameters settings become a serious issue. Even more so when mutation rates are controlled over time, as we do it in our work.

In the above-referenced works, Genetic algorithms (GA) have been the favored optimization technique. Another popular optimization technique has been Particle Swarm Optimization(PSO) [14, 17, 19, 20]. Although most works have focused on fixed settings of the signals, there are also important works on dynamic settings, for example [15, 16, 18, 22, 23].

In our work we focus on a real-world network that covers a wide area of 40 Km^2^ and include in its main backbone seventy signalized intersections, a relatively large number similar to [8] and [21]. We consider a different offset and a different cycle length per signal, instead of a common cycle for all signals. The traffic simulation is agent-based at a microscopic level, and mobility is modeled based on agents’ activities, which allows us to build and test real world scenarios for the city under study. Our study also includes analyses of solutions to understand the systemic effects of the optimization based on several indicators that measure the sustainability of transport systems [24].

## Method

### Components and overview

**Fig 1:**
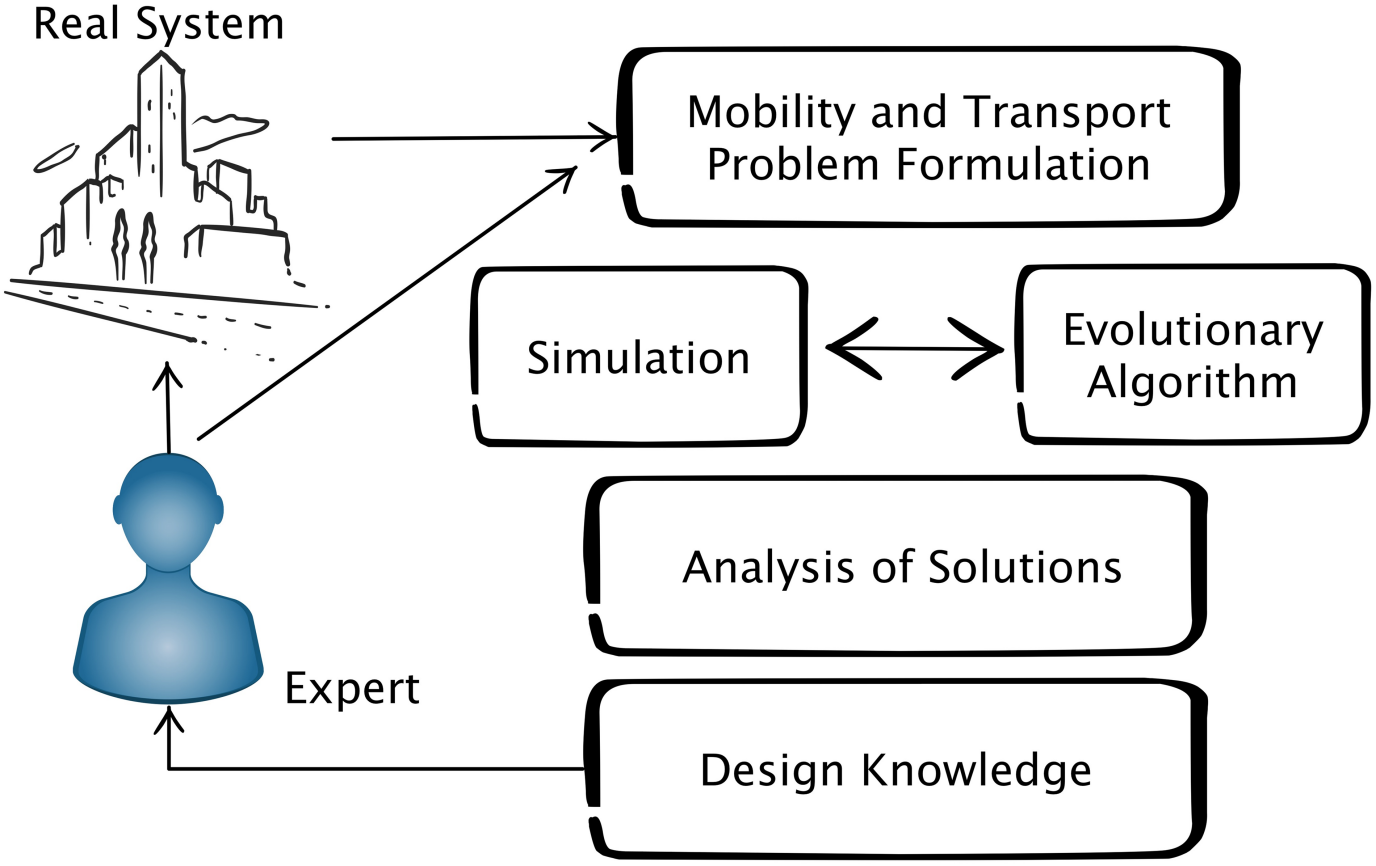
Method.

We follow the design optimization approach illustrated in Fig 1. We first formulate a problem related to mobility and transport, simulate the mobility in the city using a specialized transport simulator, use an evolutionary algorithm to find optimal solutions to the formulated problem evaluating the quality of solutions using the outcome of the simulation, analyze the solutions produced by the evolutionary algorithm and extract knowledge about the system. The approach is iterative, where the solutions and obtained knowledge are made available to the expert for further analysis. The feedback from the expert could be used to reformulate the problem to study additional details. Once the expert is satisfied, the knowledge gained could be suggested to improve the real system.

### Problem formulation

In this study we simulate the mobility of a large number of agents and study the impact of traffic light settings on travel time, fuel consumption and emissions. Besides, we analyze optimal solutions to get patterns of signal settings and verify these patterns according to their geolocation. The geographical area of study is a large and important part of Quito (Ecuador). It includes the business district, eight major universities, several hospitals, large malls, two large parks, and one major soccer stadium, covering approximately 5 x 8 Km^2^. Most signalized intersections in the area of study are two-phased and implement a fixed-time control. Only a few main intersections use left turn signals because most intersections where left turns are allowed have been gradually redesign to include diverging and merging ramps. Also, there are exclusive lanes for some bus lines, but there are no signals with special phases to establish bus transit priority. Recently there are efforts to study the incorporation of some adaptive signals. Since several sectors of the city are highly congested at certain hours, it is anticipated that adaptive signals would be only partially effective. Thus, its is important to have fixed-time optimal schemes that can be used as default timing plans to improve traffic conditions in the city. In Quito city, mobility represents a constant challenge due to the transportation infrastructure is not adequate to the demand. Quito’s urban population increased from 1 million in 1990 to 1.7 million in 2015, and it is expected to increase other 0.5 million by 2030 [1]. Similarly, in Ecuador, the rate of vehicles for each 1000 inhabitants changed from 17 to 51 from 1990 to 2010 [25]. The re-design of road network infrastructure implies substantial costs that are hardly affordable. Hence, a way to reduce traffic congestion is to make better use of the existing road network, which can be achieved in part by a proper set of traffic signals. Furthermore, proper setting of traffic signals also helps reducing emissions and can induce traffic patterns to control speed in sensible areas to increase pedestrian security.

### Transport simulator

In this work, we use MATSim, an agent-based transport simulation framework [2]. It performs a micro-simulation of agents that move on a transport system producing information about agent routes and movements. MATSim requires as inputs the model of the transport network infrastructure and the agents’ mobility plans.

The network information is obtained from Geofabrik and OpenStreetMap [26]. The number of links of the network corresponding to the area of study is 8192. The links’ attributes are length (m), flow capacity that defines how many cars can pass through the road during a unit of time, e.g. vehicles/hour, and free speed that represents the maximum flow velocity. For this work, we take into account all the main and secondary pathways with free speeds equal or above 30 Km/h. Traffic in all main pathways is bidirectional, and some of them include multiple lanes. Traffic in secondary pathways is mostly unidirectional.

One or more trips define the mobility plan of an agent. MATsim receives the *initial* mobility plans for all simulated agents, compute initial routes based on heuristics, and optimize [27] the departure time and the routes in order to provide a system in equilibrium state [28], where no traveler can improve his/her utility function by unilaterally changing departure times or routes. The configuration of initial plans is detailed in section Initial Mobility Plans for MATSim as part of the Experimental Setup.

**Fig 2:**
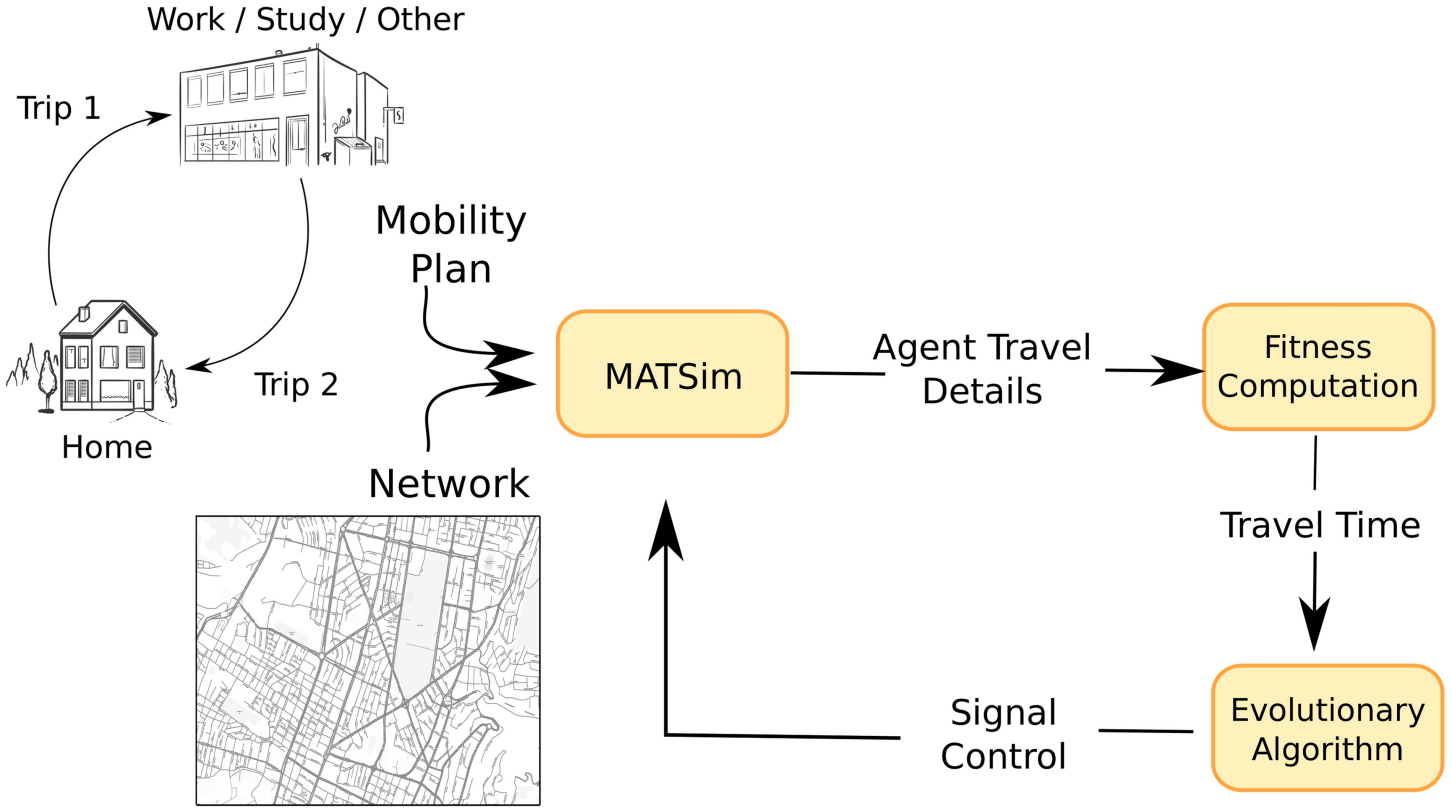
Simulation and EA integration.

Fig 2 shows the integration between the simulator and the evolutionary algorithm (EA). The EA searches optimal settings for the traffic lights. To evaluate a solution, MATSim simulates the mobility of all agents starting from the equilibrium state computed previously and setting its signals controls with the tentative solution provided by the optimizer. MATSim simulates traffic lights microscopically using fixed-time controls [29]. The output collected from that iteration of the simulator is used to calculate travel time and passed back to the optimizer as the fitness of the solution.

### Evolutionary algorithm

The optimizer is an evolutionary algorithm that combines elitism with varying mutations. In the following, we detail the representation, the flow of the algorithm, operators, the varying mutation schedule, and fitness function.

#### Traffic signals representation

The principal components of a traffic signal are cycle length, phase, offset, green and inter-green time. *Cycle length* is the time in seconds required for one complete color sequence of the signal. A *phase* is the set of movements that can take place simultaneously. An *Offset* is the time lapse in seconds between the beginning of a corresponding green phase at an intersection and the beginning of a corresponding green phase at the next intersection. In this work we extend the representation used in [8] to include a cycle per signal. Also, we use integer instead of binary representation. The variables per signal to optimize are cycle length, offset, and green times. We choose these three variables because we aim to find appropriate green times for groups of signals, where signals in the same group share a similar cycle length and are coordinated with the offset but different groups use different cycle lengths. Thus, a solution ***x*** with the specification of all *n* signals considered in the system is represented by

$$x=(S_{1},\cdots ,S_{h},\cdots ,S_{n}),$$ (1)

where the *h*-th traffic signal is defined by the following tuple of integer variables

$$S_{h}=(C_{h},\theta_{h},\phi_{h,1},\cdots ,\phi_{h,r_{h}}).$$ (2)

**Fig 3:**
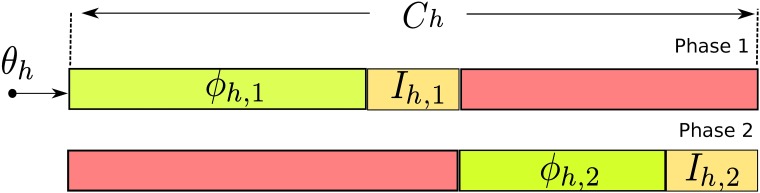
Traffic light components.

**Fig 4:**
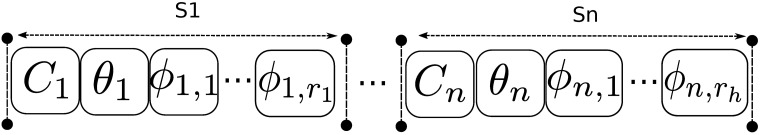
Solution representation.

Here, *C*_*h*_ is cycle length, *θ*_*h*_ is the offset, and *ϕ*_*h*,1_,⋯, *ϕ*_*h*,*r*_*h*__ are the green times for the *r*_*h*_ phases of the signal. Fig 3 illustrates the main components of a signal and Fig 4 the representation of a solution to a system with *n* signals, each one with *r*_*h*_ phases.

The ranges and constraints of these variables are given in Eqs (3)–(9), where *I*_*h*,*j*_ is the inter-green time at signal *h* for phase *j* and *r*_*h*_ is the total number of phases of signal *h*. Equations Eqs (3)–(5) represent the range for cycle length *C*_*h*_, offset *θ*_*h*_ and green time *ϕ*_*h*,*j*_, respectively. *C*_*min*_ is determined by identifying the signal that needs the longest duration just to accommodate the inter-green times and the minimum green times as shown in Eq (6). *C*_*max*_ is set to 135 seconds and inter-green per phase to 3 seconds. These values imply that the minimum cycle time *C*_*min*_ is 40 seconds in two phase signals.

The minimum green time should allow drivers to react to the start of the green interval and meet driver expectancy. We follow the Traffic Signal Timing Manual (TSTM) [30] guidelines taking into consideration the driver expectancy [31] and pedestrian crossing time. TSTM suggests a minimum green time to satisfy driver expectancy between 7 to 15 seconds for major arterials with speed limit of 64 Km/h or less. In our scenario the free speed in most major arterials is around that value. As we mentioned in Problem Formulation section, the business district has several facilities and services with a high pedestrian volume in some areas. Thus, considering both, the driver expectancy and walk interval duration for pedestrian crossing, we decided to set the minimum green time to 17 seconds for all signalized intersections as shown in Eq (7).

Regarding the maximum green time, TSTM suggests between 40 to 60 seconds for the kind of arterials considered in our scenario. In our work, we search simultaneously cycle length, green times and offsets. As mentioned above the range allowed for cycle length is between 40 and 135 seconds. The maximum cycle length (135) allows us to set maximum green times (as suggest by TSTM) to both phases.

Eq (8) ensures that the sum of the green times in a signal together with inter-green do not exceed the cycle length set for the signal. Eq (9) establishes the maximum green time for the signal phase based on the cycle time, inter-green and minimum green time.

$$C_{min}\;\leq \;C_{h}\;\leq \;C_{max}\;$$ (3)

$$0\leq \theta_{h}\leq C_{h}-1$$ (4)

$$\phi_{{h,j}_{min}}\leq \phi_{h,j}\leq \phi_{{h,j}_{max}}$$ (5)

$$C_{min}=\underset{h=1,2\ldots ,n}{\text{max}}\{(\sum_{j=1}^{r_{h}}\phi_{{h,j}_{min}}+\sum_{j=1}^{r_{h}}I_{h,j})\}$$ (6)

$$\phi_{{h,j}_{min}}=17\;sec\;\forall h,j$$ (7)

$$C_{h}=\sum_{j=1}^{r_{h}}\phi_{h,j}+\sum_{j=1}^{r_{h}}I_{h,j}\;\forall h$$ (8)

$$\phi_{{h,j}_{max}}=C_{h}-\sum_{j=1}^{r_{h}}I_{h,j}-\sum_{k=1,k\neq j}^{r_{h}}\phi_{{h,k}_{min}}$$ (9)

#### Evolutionary algorithm flow

**Fig 5:**
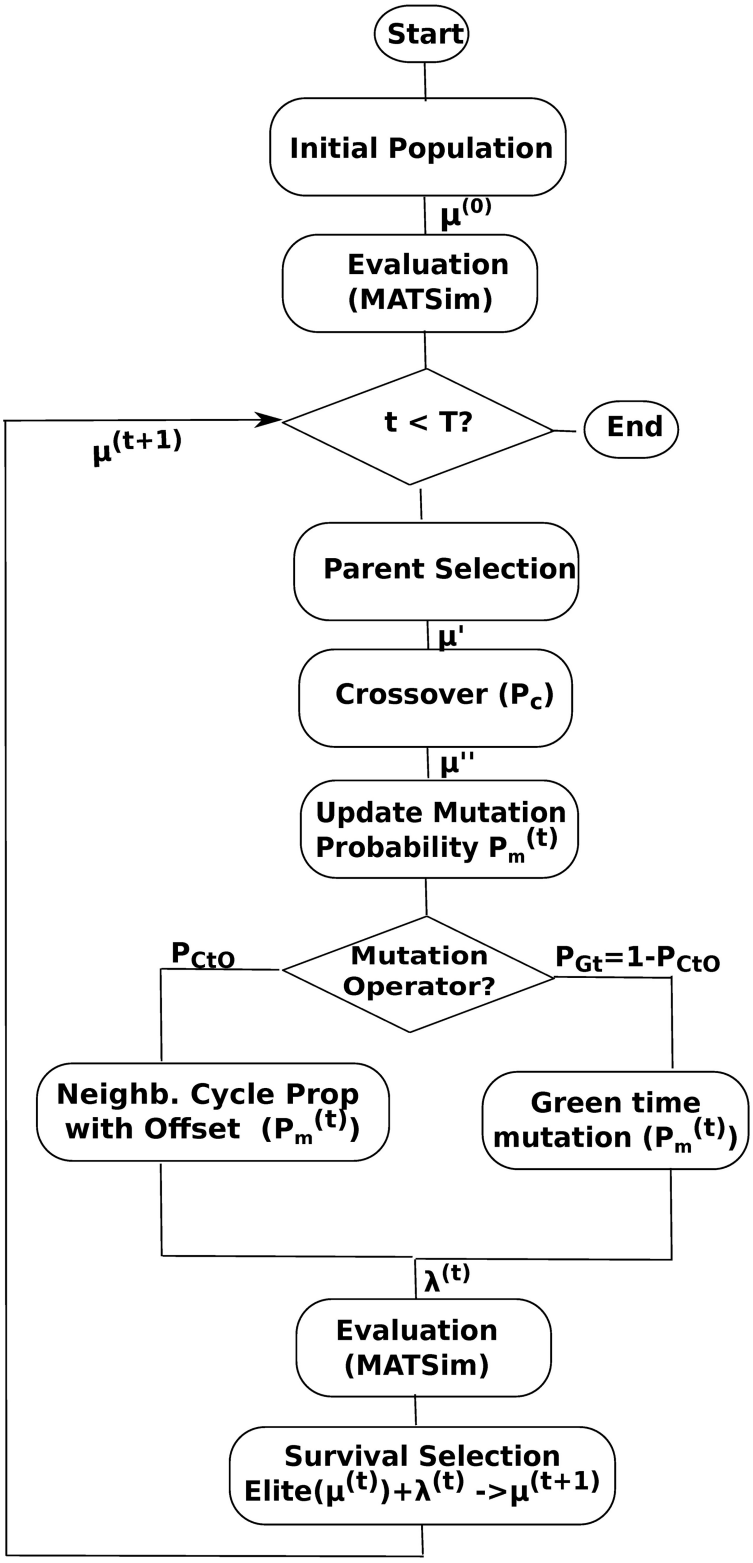
Evolutionary algorithm flow chart.

A block diagram that illustrates the flow of the algorithm is shown in Fig 5. In the following, we describe the main steps of the algorithm based on this figure.

The initial population *μ* is formed by 20 deterministically created solutions. All signals in a solution are set to the same cycle length, green times are set evenly for all phases dividing cycle length minus inter-greens by the number of phases, and offsets are set to zero. In this work, we consider signals with two phases. Solutions in *μ* are assigned different cycle lengths, from 40 to 135 seconds in steps of 5. No repetitions are allowed. We do this to guarantee maximum diversity for each signal across the population so that, evolution could explore a broad region of variable space.

Each solution is evaluated running MATSim with the traffic signals settings it encodes. Parents are selected for reproduction using binary tournaments among randomly sampled solutions. The offspring population λ is created applying crossover to the selected parents with probability *P*_*c*_ followed by mutation. Mutation updates the probability $P_{m}^{\left(t\right)}$, selects a mutation operator and apply it with the updated $P_{m}^{\left(t\right)}$ per signal. There are two mutation operators, one for cycle time and offset and another for green time. The operators’ probabilities are *P*_*CtO*_ and *P*_*Gt*_ = 1 − *P*_*CtO*_, respectively. During the evolution we relax the initial assumption of common cycle length for all signals in a solution. The proposed mutation operators for cycle time and offset searches for groups of signals with same cycle length by propagating the cycle of a signal to its neighbors and induce their coordination by setting their offset based on the distance between neighboring signals. The operators’ probabilities allows to balance the search for cycle lengths and green times.

After crossover and mutation, the offspring is evaluated running MATSim as indicated above. Selection of solutions for the next generation applies elitism, taking into consideration the current population *μ*^(*t*)^ and the offspring population λ^(*t*)^. Namely, the population *μ*^(*t*+1)^ is formed by the best in the combined population of the top *nElite* from *μ*_*t*_ and the λ_*t*_ offspring, i.e. *nElite*(*μ*^(*t*)^) + λ^(*t*)^ → *μ*^(*t*+1)^. Also, we introduce varying mutation rates for $P_{m}^{\left(t\right)}$. The algorithm evolves solutions repeating parent selection, crossover and mutation, offspring evaluation, and survival selection until a pre-determined number of generations *T* has been reached. The appropriate combination of operators, high selection pressure via elitism, and varying mutation rates accelerate convergence of the algorithm and allows to search effectively even with small populations.

#### Operators of variation

In this work, we use either one or two point crossover to interchanges signals between parents. The crossing points are selected randomly with equal probability in the range [1,*n* − 1], where *n* is the number of signals.

The algorithm selects between two operators to mutate signals. One is the *Green time mutator (GtM)* and the other one is *Neighborhood cycle propagation with distance-based offset mutator (NCtOPM)*.

The *GtM* operator decreases the green time of one phase and adds it to another phase using step size *stepGt*. The phase *i* to decrease its green time is randomly chosen among those where the decrement does not violate the constraint for minimum green time *ϕ*_*h*,*i*_*min*__. Similarly, the phase *j* ≠ *i* to increase its green time is also randomly chosen.

**Fig 6:**
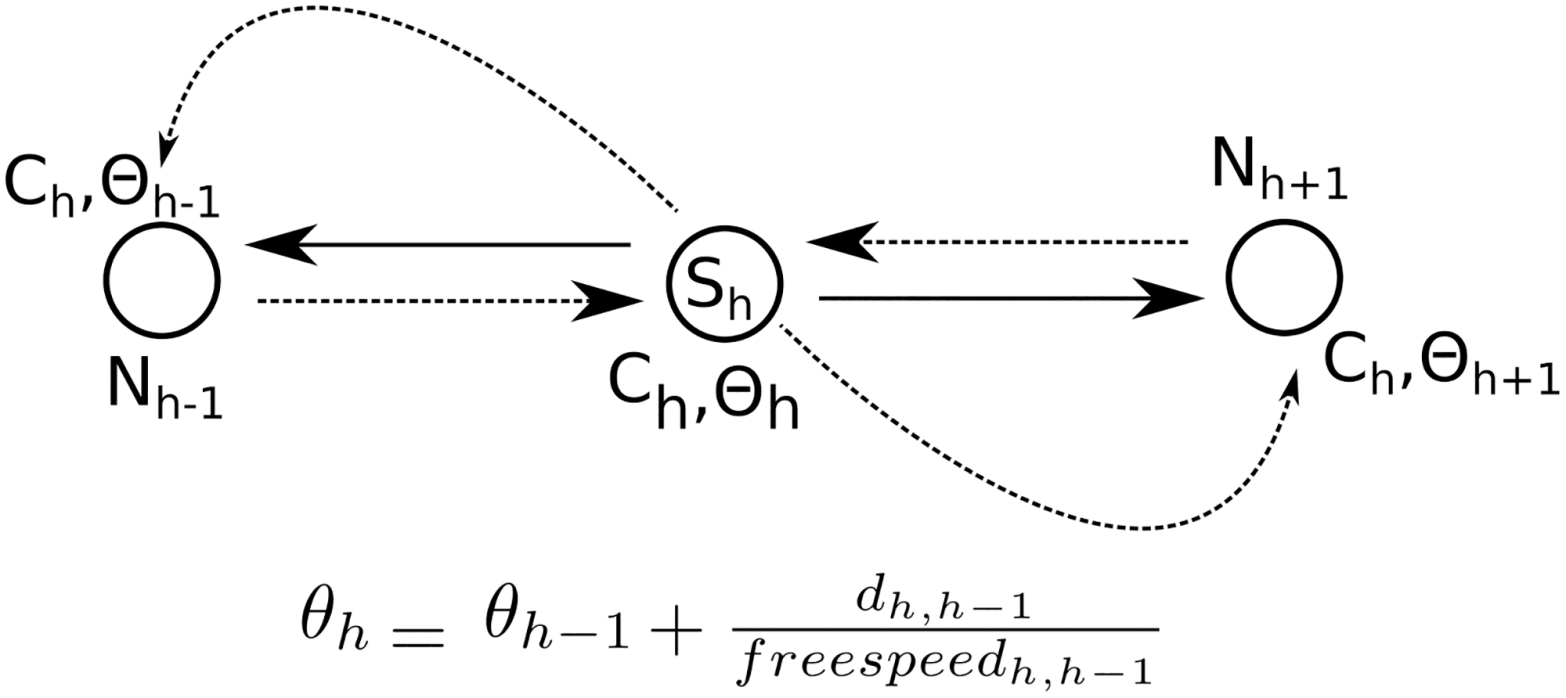
Cycle and offset propagation.

The *NCtOPM* operator aims to improve traffic flow along the two main axis of circulation, South-North-South (SNS) and West-East-West (WEW), favoring traffic signal coordination by simultaneously modifying the parameters of a signal *S*_*h*_ and its neighbors *N*_*h*_. The operator stochastically selects the axis of circulation, either NSN or WEW. Then, it chooses one direction of the axis, for example, NS for axis NSN, and propagates the cycle length (*C*_*h*_) of the reference signal (*S*_*h*_) to its neighbors in that direction. In addition, it sets the offsets of the neighboring signals based on the time required to cover the distance *d* from *S*_*h*_ to the neighbor traveling at free speed. The operator does the same with the other direction of the axis, i.e. SN. The propagation of cycle and offset to the neighborhood is illustrated in Fig 6. Since the cycle length of the signals may change, green times are also adjusted so that the ratios of green time per phase to cycle are the same before and after propagation.

#### Varying mutation schedule

Varying mutation operators combined with high selection pressure have been proved effective to accelerate the convergence of evolutionary algorithms [32, 33]. The proposed algorithm introduces elitism inducing a high selection pressure. Also, it includes a time-dependent schedule that deterministically varies mutation rate $P_{m}^{\left(t\right)}$ in the range $\left[P_{m}^{\left(0\right)},P_{m}^{\left(T\right)}\right]$. $P_{m}^{\left(t\right)}$ varies in a hyperbolic shape [32] and is expressed by

$$P_{m}{}^{\left(t\right)}={\left(\frac{1}{P_{m}{}^{\left(0\right)}}+\frac{\frac{1}{P_{m}{}^{\left(T\right)}}-\frac{1}{P_{m}{}^{\left(0\right)}}}{T-1}t\right)}^{-1}$$ (10)

where *T* is the maximum number of generations, *t* ∈ {0, 1, …, *T* − 1} is the current generation, ${P_{m}}^{\left(0\right)}$ and ${P_{m}}^{\left(T\right)}$ are the desired mutation probabilities per signal at time 0 and *T*, respectively.

#### Fitness function

The fitness of a solution is the average travel time of all agents computed from MATSim’s simulation, setting traffic lights with the values encoded in the solution. MATSim outputs, for all agents, the time it takes to travel each link of an agent’s route. The fitness function is expressed by

$$\text{min}\;\frac{\sum_{i=1}^{V}\sum_{l=1}^{L}t_{il}}{V},$$ (11)

where *t*_*il*_ is the travel time on link *l* for vehicle *i*, *V* is the number of vehicles being simulated, and *L* is the number of links in the network.

## Experimental setup

### Evolutionary algorithm

We run the algorithm ten times per experiment, use each time a different random seed but always start from the same initial population. We configure three experiments named E3, E4, and E4DVM to test different strategies with mutation operators. The experiments are described with more detail in section Effects of Operators.

The number of generations is set to 50, population size is 20, and the number of elite individuals is *nElite* = 10. Unless stated otherwise, crossover rate is set to *P*_*c*_ = 1.0 and the range for varying mutation per signal $P_{m}^{\left(t\right)}$ is [20/*n*, 4/*n*], where *n* = 70 is the number of signals. To compare with varying mutation probability per signal, we also apply the mutation operators with constant probability *P*_*m*_ = 4/*n* per signal. Probabilities *P*_*CtO*_ and *P*_*Gt*_ of the mutation operators *GtM* and *NCtOPM* are detailed in Table 1 together with the mutation probabilities per signal for the three experiments.

**Table 1:** Probability of mutation operators *P*_*CtO*_ and *P*_*Gt*_ and mutation probability *P*_*m*_ and $P_{m}^{\left(t\right)}$ per signal.

Exp	Op.Prob *P*_*CtO*_, *P*_*Gt*_	Mutation Probability per Signal	Observations
E3	0.7,0.3	*P*_*m*_ = 4/70	Constant
E4	0.3,0.7	*P*_*m*_ = 4/70	Constant
E4DVM	0.3,0.7	$P_{m}^{\left(t\right)}=\left[P_{m}^{\left(0\right)}=20/70,P_{m}^{\left(T\right)}=4/70\right]$	Varying

Step size for the green time mutation operator *GtM* is *stepGt* = 3. In the case of the neighborhood mutation operator *NCtOPM*, we define in advance two neighborhoods for each signal *S*_*h*_, one for the axis of circulation SNS and another one for the axis WEW. The neighborhoods are based on the actual geographical location of the signals. Also, we pre-calculate the distance and the average free speed to its neighbors along each axis. In this work, the radius of the neighborhood is set to one, i.e. the neighborhood includes the next and previous signal to the reference signal. The neighborhood operator selects for mutation the axis NSN and WEW with probabilities 0.85 and 0.15, respectively, in agreement with the most common traffic flow and mobility patterns in the city.

### Initial mobility plans for MATSim

To simulate the movement of the agents, we need to provide MATSim with their initial mobility plans. In the following, we explain in detail how these plans are modeled. In this work, a mobility plan of an agent consists of two trips. In the first trip, the agent leaves home towards the location of its activity [34] and in the second trip, the agent returns home from its activity. Thus, each trip is defined by the coordinates of origin and destination of the trip and the activity specified by the type, starting time and duration of the activity. The type of vehicle used is also associated with the journey.

**Fig 7:**
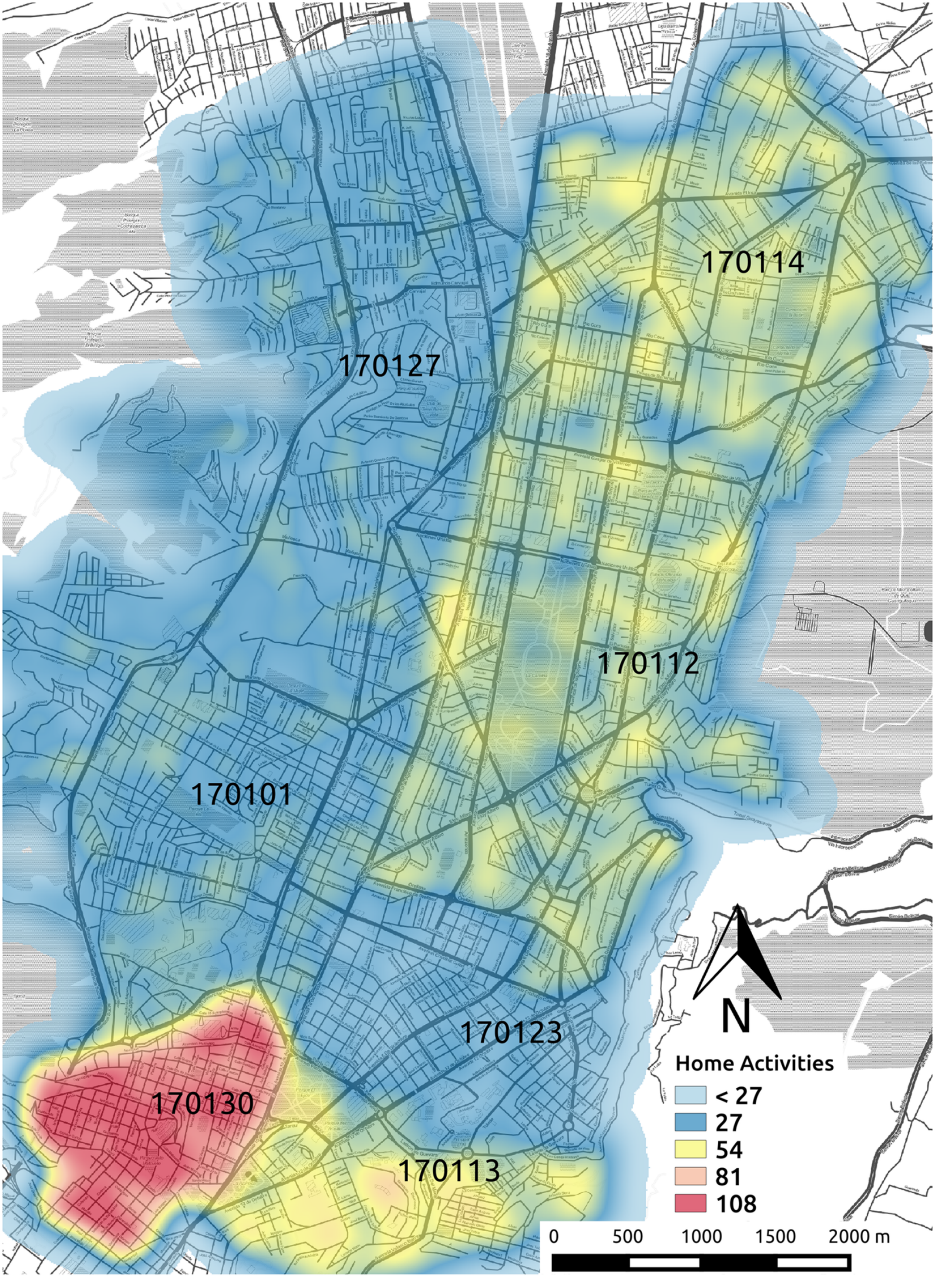
Distribution of home coordinates.

**Fig 8:**
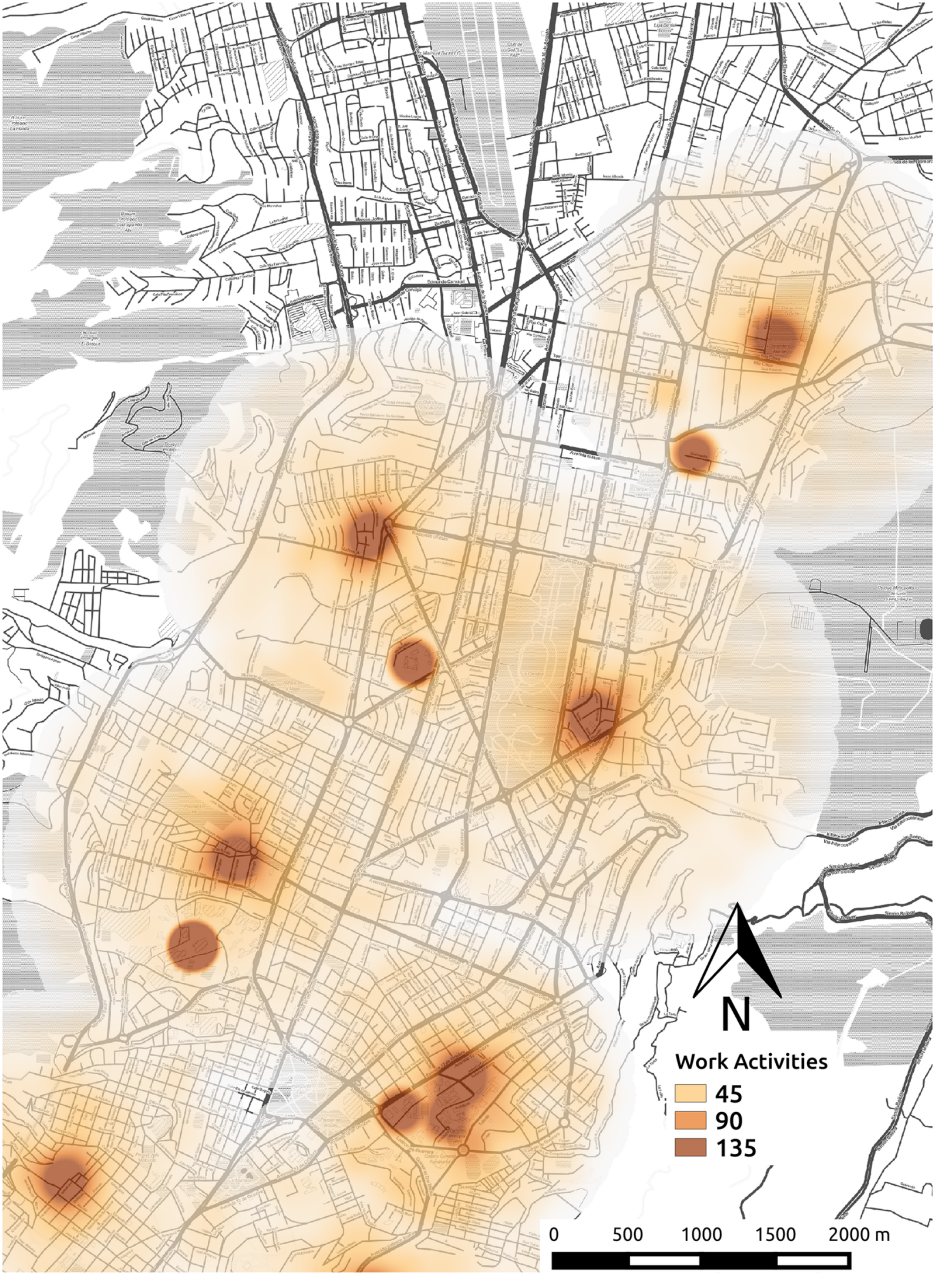
Distribution of main activities coordinates.

We digitalized the administrative city borders defining a geographical area per district using QGIS Geographic Information System [35]. We sample home locations for the agents from these districts in proportion to the actual population. We consider three types of activity: *work*, *study*, and *others* for activities such as leisure, business, shopping, and so on. The proportions of agents for these activities are 32%, 33% and 36% respectively, according to mobility survey data [36]. Location coordinates of activities are determined based on actual information about the city. In the case of activity *work*, data about the distribution of workplaces by district in Quito is provided in [37]. Fig 7 shows in colors the distribution of the home coordinates of the agents, Fig 8 shows the coordinates of the main activities, and Table 2 the population of the districts considered in our simulation.

**Table 2:** Population per District (Census 2010 [38]).

code INEC	District	Inhabitants
170101	Belisario Quevedo	45.370
170112	Iñaquito	44.149
170113	Itchimbia	31.616
170114	Jipijapa	34.677
170123	Mariscal Sucre	12.976
170127	Rumipamba	31.300
170130	San Juan	54.027
	Total	254.115

We follow that distribution to assign the coordinates of workplaces probabilistically. For the type of activity *study*, we select probabilistically among the universities located in the area of study and assign its coordinates. The number of agents assigned to each university is proportional to the university’s population. In this activity, we neither consider elementary nor high school students. For the type of activity *other*, we use an origin-destination probability matrix to assign the coordinate, where the probability to go to a destination is proportional to the population of the destination district and inverse to the distance.

Starting times and durations of activities are assigned randomly sampling from ranges defined for each type of activity.

**Fig 9:**
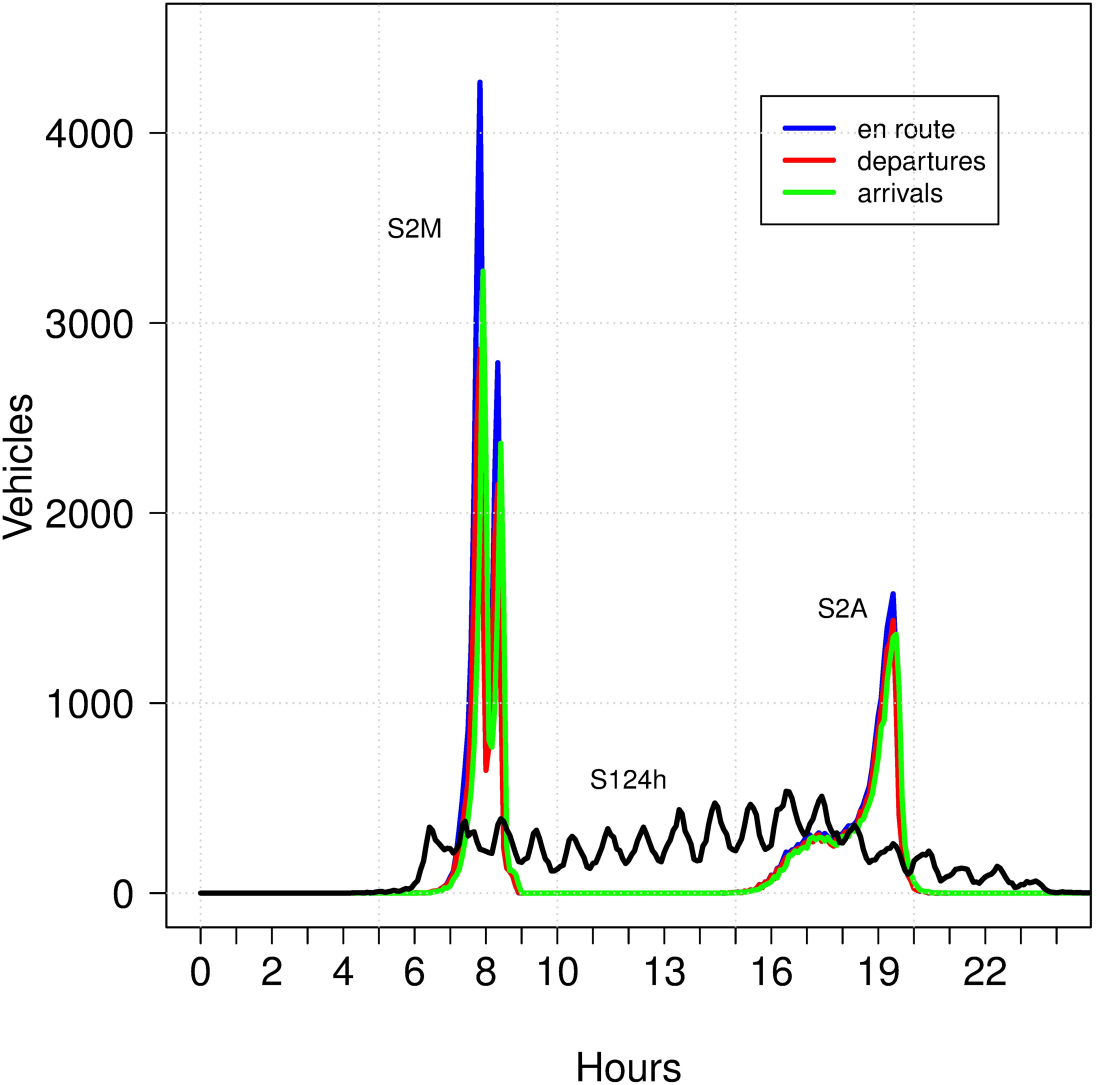
Mobility scenarios S124h, S2M and S2A.

In this work, we design three different mobility scenarios as illustrated in Fig 9. In the first one (*S124h*), the trips start from 06:00h and there are several small peaks during the whole day where 500 vehicles or less are in route. In the second one (*S2M*), the agents move during morning hours (06:00h-09:00h) from home to their activity destinations, with a high peak between 07h30 and 08h30 where more than 4000 vehicles are in route. In the last one (*S2A*), the same agents of *S2M* move back home. In S2A the trips are spread over a longer period from 15h00 to 20h00 with a peak around 19h00, where approximately 1500 vehicles are in route. These mobility plans of *S2M* and *S2A* are conceived to study the system under saturated scenarios during morning and afternoon peak hours.

**Fig 10:**
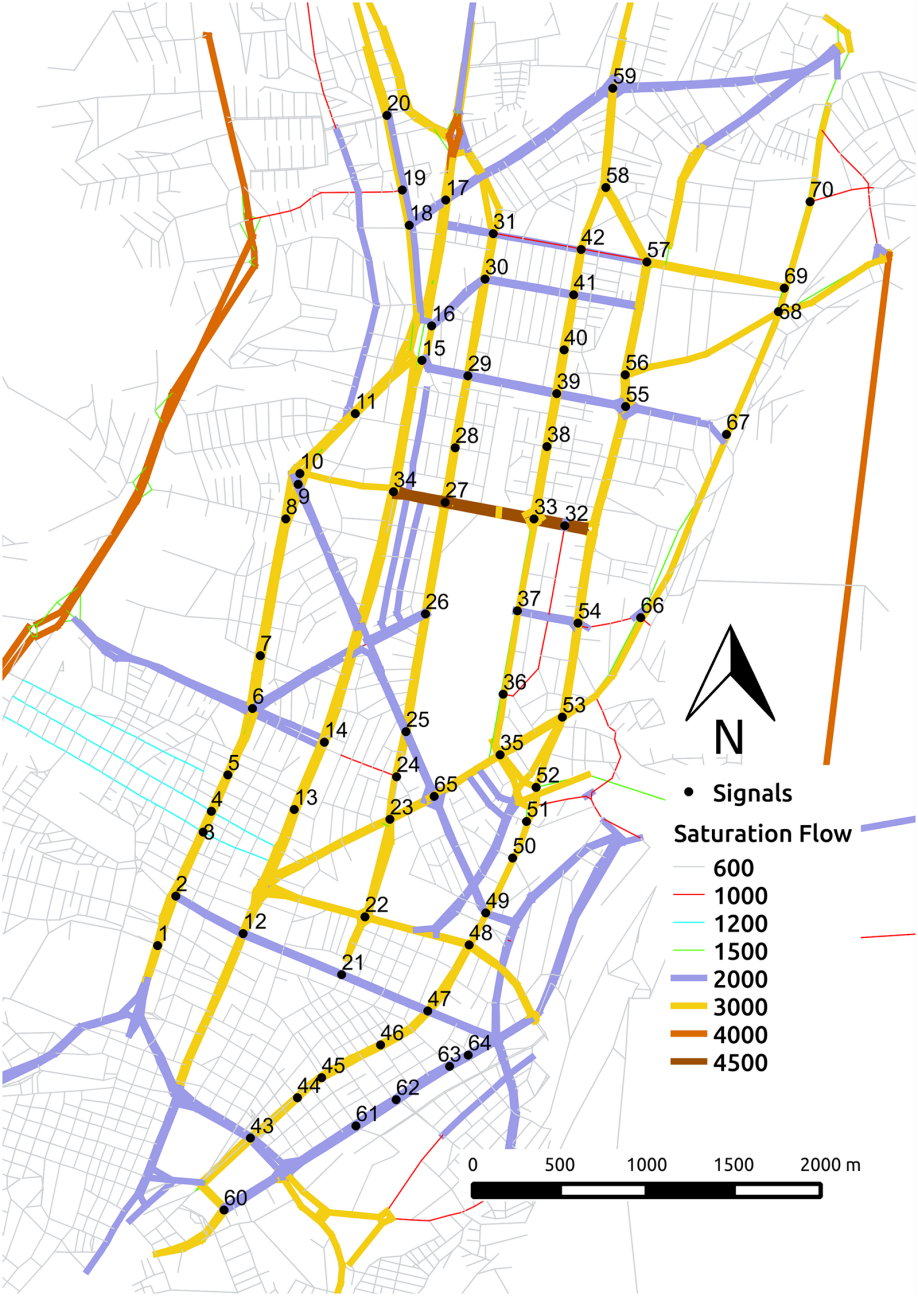
Saturation flows.

Fig 10 shows the saturation flow rates, i.e. number of passenger car units (PCU) per hour, of lane groups of the network links. One-lane links are shown in gray and red, two-lane links in blue, green, violet, and orange, and three-lane links in brown. Thus, the saturation flow per lane are between 600 and 1500 PCU per hour, which is less than the base saturation flow of 1900 assumed by the The Highway Capacity Manual [39].

The three scenarios simulate the mobility of 20.000 agents. Thus, the less saturated scenario is *S124h*, and the most saturated one is *S2M*. This number of agents represents approximately the 30% of the estimated number of vehicles according to the inhabitants in the zone of study [36]. We assign a type of vehicle to each agent as explained in section Emissions and Fuel Consumption. MATSim requires around 8 hours to reach the equilibrium state of 20.000 agents and around 2.5 minutes per individual to compute its fitness.

The average number of vehicles is around 3,660 vehicles/hour in the morning rush hours from 7:00 to 9:00 am and 3,655 vehicles/hour in the afternoon hours from 15:00 to 20:00 according to traffic count data taken in 8 intersections located in the area of study. As indicated above, our scenario *S*2*M* simulates a saturated situation that approaches the counters observed in the morning rush hour. The other two scenarios model less saturated situations to test signal settings under different conditions.

## Simulation results and discussion

### Effects of operators

In this section, we study the effects of the operators of variation. First, we fix one point crossover and look at mutation operators applied with constant or varying mutation probability.

**Fig 11:**
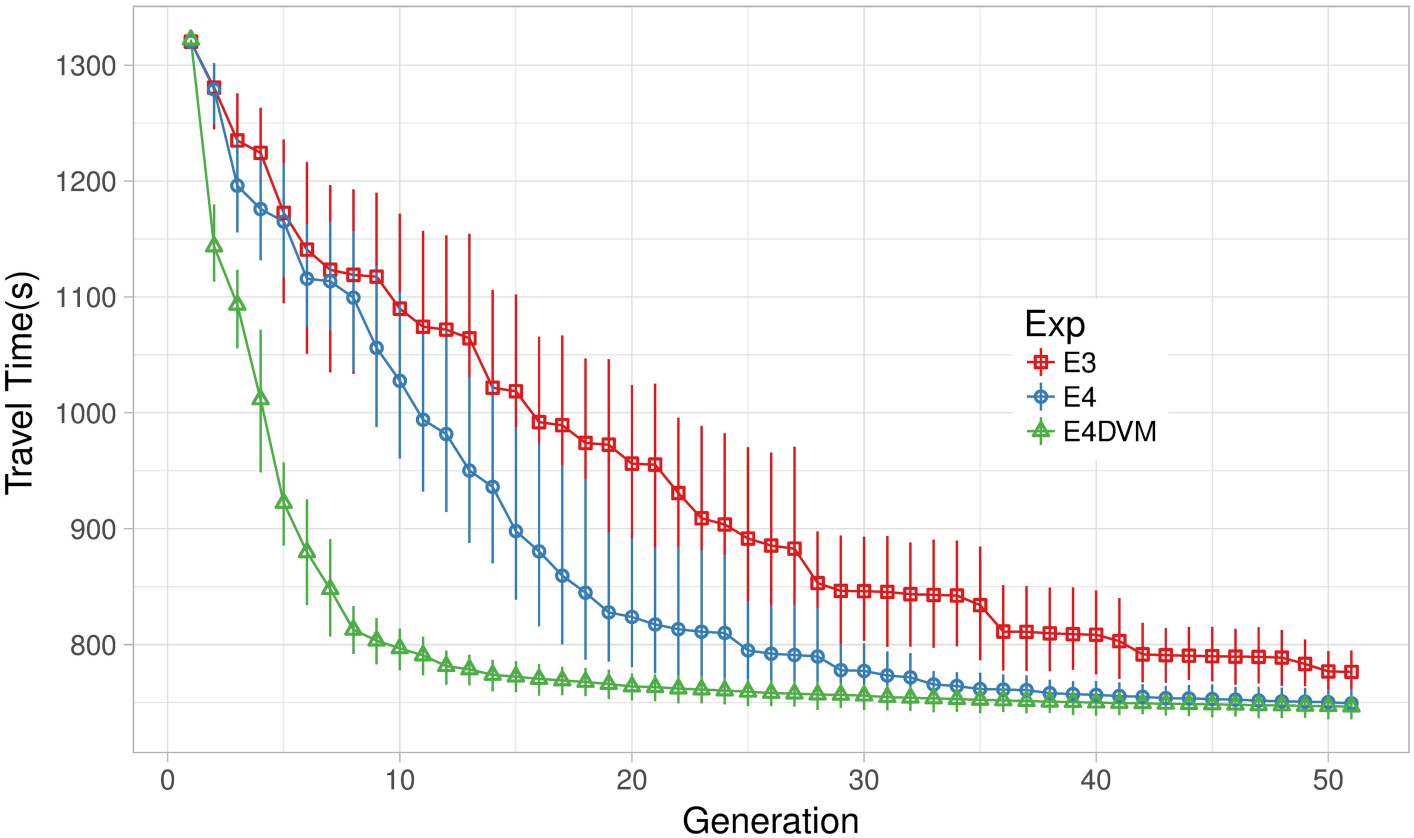
Mean travel time over generations in experiments E3,E4 and E4DVM.

**Fig 12:**
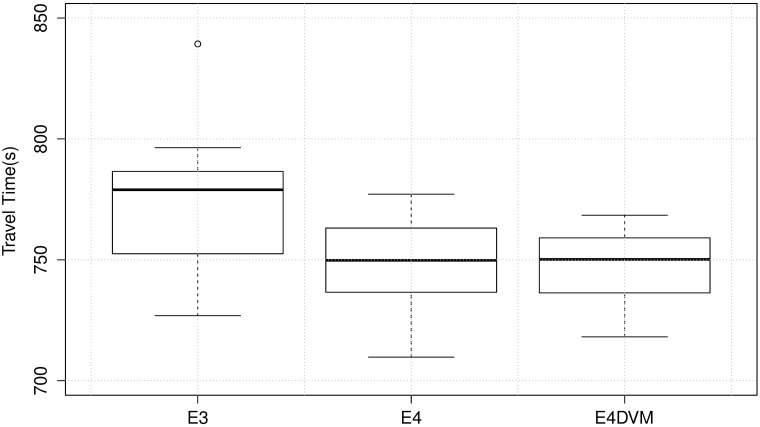
Mean travel time of best solutions in experiments E3,E4 and E4DVM.

Fig 11 shows the mean travel time over the generations for mobility scenario S124h. In addition, variance of the best solutions at the last generation is shown in detail in Fig 12 and Table 3 includes numerical values of standard deviation and inter-quartile range.

**Table 3:** Mean travel time, standar deviation and interquartile range of best solutions in experiment E3,E4 and E4DVM.

Exp.	Travel Time		
	avg	std	iqr
E3	776.44	30.64	34.04
E4	749.34	20.36	26.53
**E4DVM**	**746.50**	**17.43**	**21.20**

Experiments E3 and E4 apply the *NCtOPM* and *GtM* operators with constant mutation probability *P*_*m*_ = 4/*n* per signal. E3 applies more often *NCtOPM* to propagate cycle and offset than *GtM* to mutate green time. Conversely, E4 applies more often *GtM* than *NCtOPM*, as shown by the operator probabilities *P*_*CtO*_ and *P*_*Gt*_ in Table 1. Note from Figs 11 and 12 that mutating more often green times (E4, *P*_*Gt*_ = 0.7) eliminates outliers and the algorithm converges to travel times lower than propagating more often cycle and offset (E3, *P*_*CtO*_ = 0.7). Also, from Fig 11 note that E3 converges slower than E4. This suggests that configurations with a relatively larger rate for the *GtM* operator to explore green times combined with lower rates for the *NCtOPM* operator to propagate cycle length and offsets lead to faster and better convergence.

**Fig 13:**
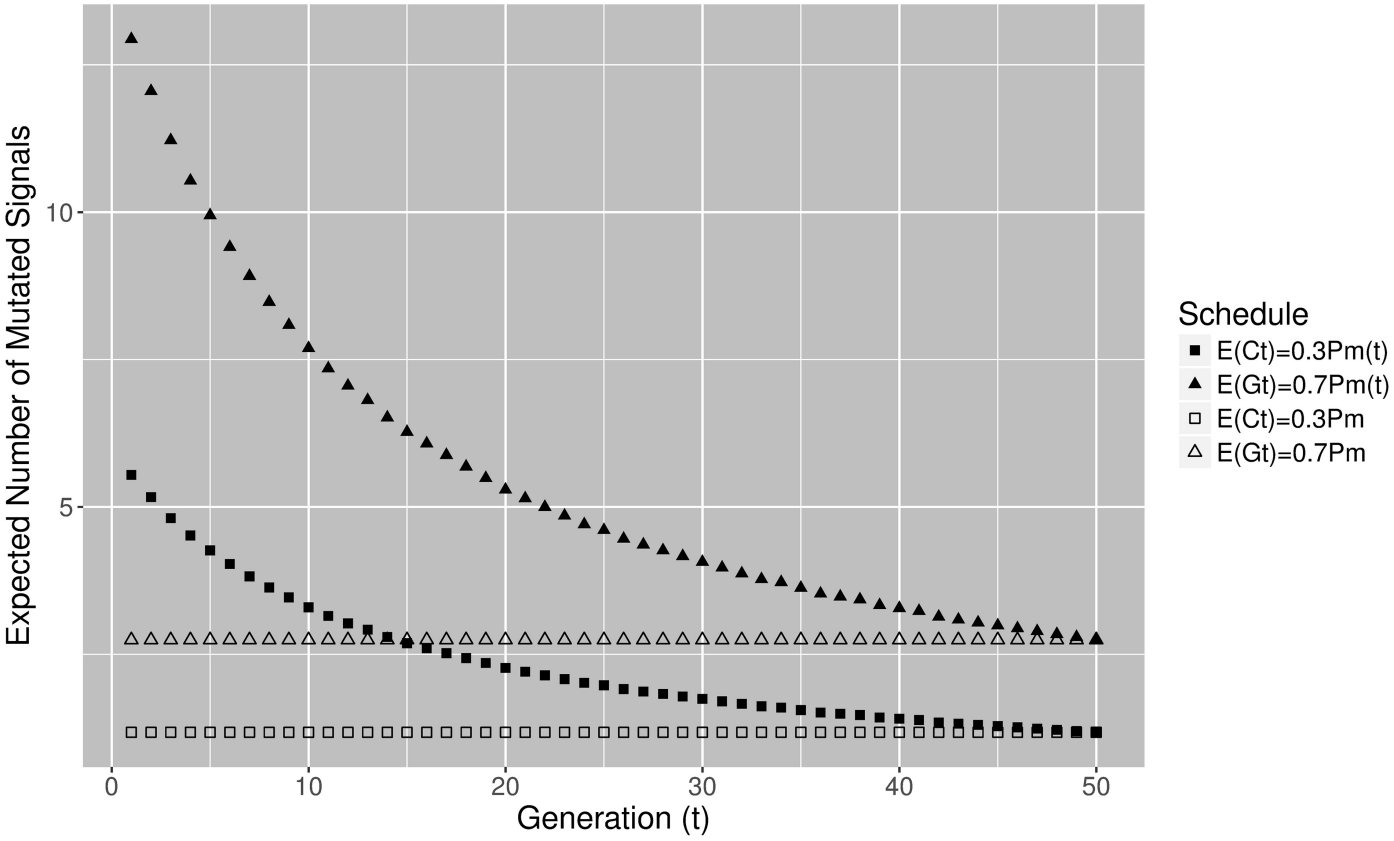
Expected number of mutated signals with constant and varying mutation schedules (E4,E4DVM).

To study whether larger mutation rates per signal could be useful, in experiment E4DVM we keep the configuration for mutation operators of E4 but instead of using constant mutation rate *P*_*m*_ = 4/*n* we vary mutation rate in the range $P_{m}^{\left(t\right)}=\left[20/n,4/n\right]$. From Fig 11 it is remarkable the fast convergence of the algorithm that applies varying mutations. Note also from Table 3 that travel time, standard deviation and interquartile range reduce further compared to E4. We also performed Mann-Whitney-Wilcoxon non-parametric tests, verifying significant statistical differences between E3 and E4 and E3 and E4DVM. Between E4 and E4DVM there is no significant difference in generation 50. This is because both algorithm configurations converge to the same good quality results given an enough number of generations. However, Mann-Whitney-Wilcoxon tests verify that there is a significant difference between generation 25 and 50 for E4 but not for E4DVM, showing that E4DVM converges faster. Fig 13 shows the expected number of mutated signals per solution by *GtM* and *NCtOPM* when constant and varying mutation are applied.

To verify the search ability and convergence of the algorithm under saturated situations, we run E4 and E4DVM configurations on denser scenarios with larger traffic volume in shorter periods of time compared to S124h.

**Fig 14:**
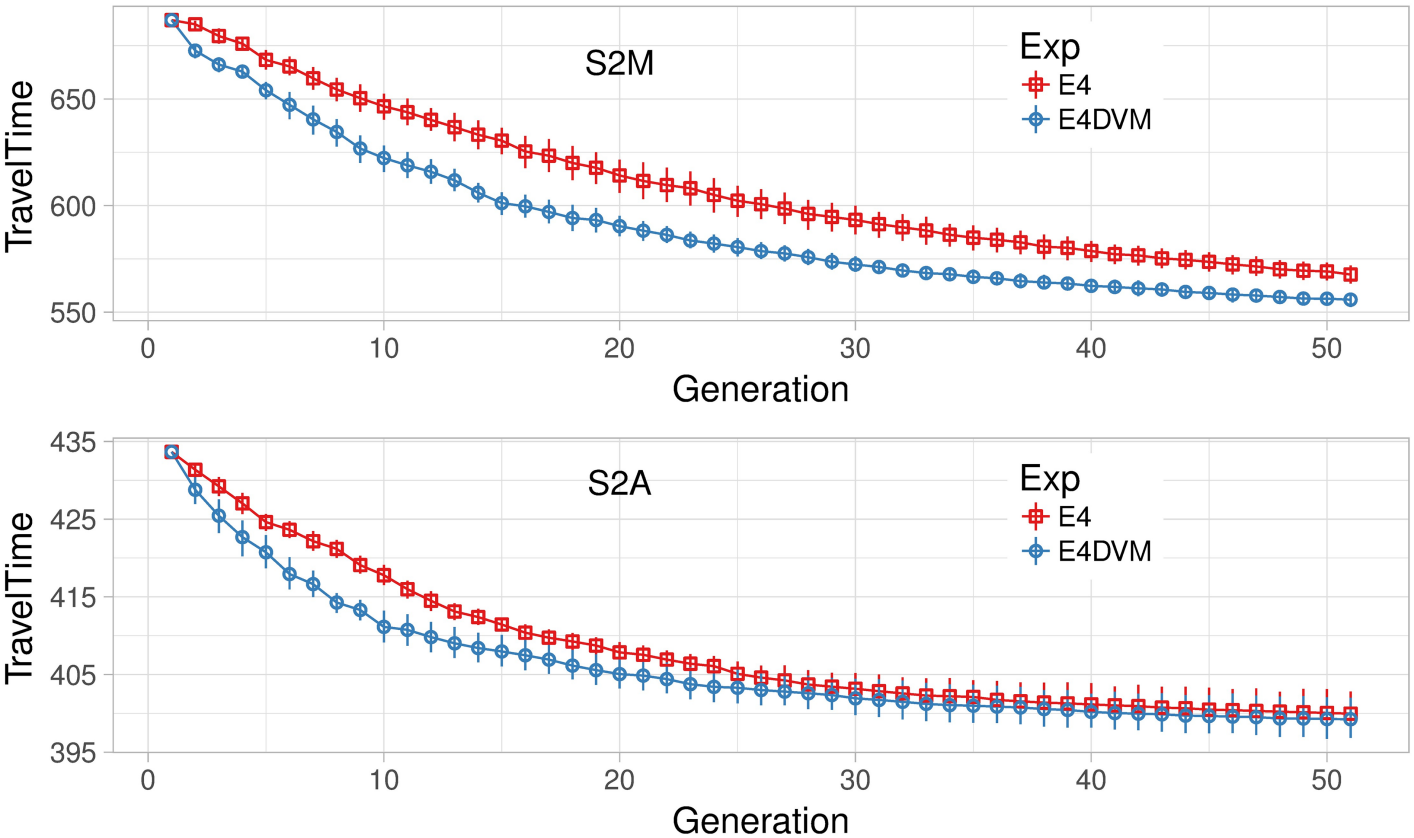
Mean travel time over generations, scenarios S2M and S2A (E4, E4DVM).

Fig 14 shows mean travel time over the generations on scenarios S2M (top) and S2A (down), respectively. From these figures it can be seen that E4DVM convergence faster towards lower travel times than E4. Additionally, it should be noted that the difference in travel time between E4DVM and E4 increases as the scenario becomes denser.

In addition to the design of appropriate mutation operators and their schedule, we also study the effects of crossover. In the above experiments we use one-point crossover with rate *P*_*c*_ = 1. We also conduct experiments setting crossover rate to *P*_*c*_ = 0 in order to switch off the crossover and replace one-point with two-point crossover keeping the same rate *P*_*c*_ = 1.

**Fig 15:**
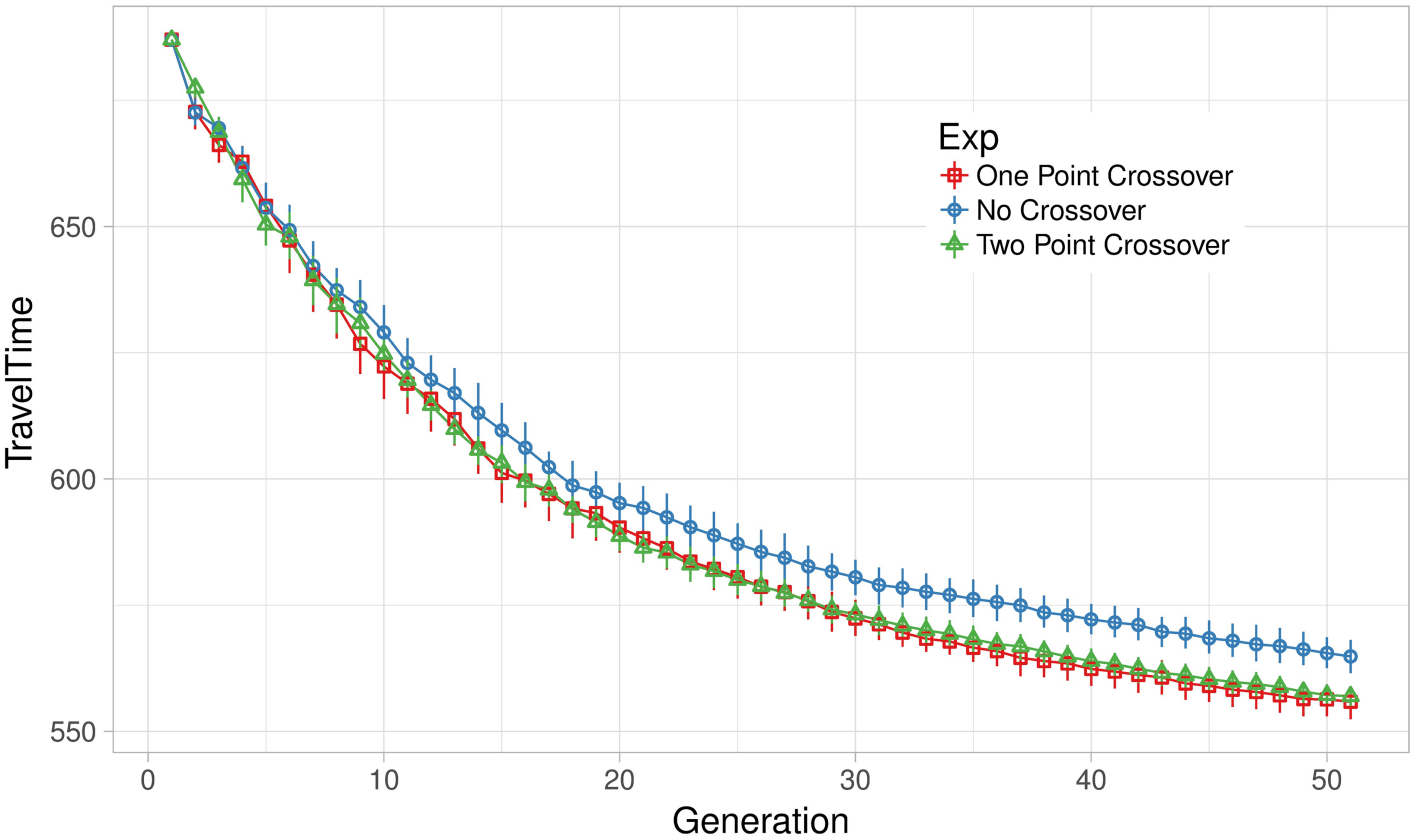
Effect of crossover operator on mean travel time.

Fig 15 shows mean travel time over generations on the densest scenario S2M by E4DVM with and without crossover. Here, it should be noted that the algorithm using either one- or two-point crossover converges to lower travel time than the algorithm that switch off the crossover. It is known that one-point crossover could be more disruptive than the two-point crossover, particularly when the landscape of the problem is rugged [40, 41]. However, note that in this scenario there is no significant difference between one-point and two-point crossover. In the future, it could be worth studying crossover operators specially tuned for this problem.

The no-crossover configuration is particularly interesting and deserves further discussion. Remember that the same value of cycle length is assigned to all signals of a solution in the initial population. Also, that different values of cycle length are assigned to different solutions. Thus, no-crossover means that signals with different cycle length never get mixed to form a new solution. In addition, the propagation of cycle length between neighboring signals in the same solution by the *NCtOPM* operator has no effect, precisely because all signals have the same cycle length. In other words, the no-crossover configuration searches only green times by the *GtM* operator and propagates offsets based on the distance between signals by the *NCtOPM* operator. This is equivalent to searching an optimal cycle length for all signals instead of one per signal. As can be seen from the figure, better solutions in terms of travel time can be found when we search an optimal cycle length per signal. Other works mostly focus on one common cycle for all signals [6, 8, 10].

The algorithm discussed here is the result of investigating several strategies and configurations of the operators. A first basic approach applies step mutation operators for the cycle and offset, in addition to the step mutation operator for green time discussed here, with an operator probability for each one of them [42, 43]. A second approach propagates the cycle length and keeps step mutation operators for green time and offset. The introduction of the neighborhood reduces variance and improves convergence speed of the algorithm over the basic approach, however, the search in offsets is not effective with a small number of generations. A third approach, included in this paper, propagates both cycle length and offset in one operator *NCtOPM* and keeps step mutation for green time. The inclusion of offset propagation allows an effective search and improves results further. Besides, we also conduct experiments applying step cycle mutation previous to propagate the cycles and offsets in *NCtOPM*. However, travel time over the generations and final results are almost the same as those obtained when cycle length is propagated without the mutation. These results suggest that the diversity of cycle lengths present in the initial population is appropriate and additional variation of cycles seem not to be required in the scenarios we study here. A detailed discussion and comparison of these approaches using constant mutation probability per signal can be found in [44]. As discussed above, another important component of the algorithm is varying mutation. Initial results comparing constant mutation per signal with the varying mutation have been reported in [45].

### Algorithm’s parameters analysis

In the previous section, we have shown that varying mutation from high to low rates per signal combined with a strong elitist selection leads to faster and better convergence. Here we use Sequential Model-based Algorithm Configuration (SMAC) [46] to derive other combinations of parameters settings related to the operators of variation in E4DVM that can lead to good performance. Namely, we analyze optimal combinations of crossover rate *P*_*c*_, the initial $P_{m}^{\left(0\right)}$ and final mutation probability $P_{m}^{\left(T\right)}$ per signal of the deterministic varying mutation schedule, and the probability of cycle and offset propagation operator *P*_*CtO*_. 1 − *P*_*CtO*_ determines the probability of the green time mutation operator. Mostly, deterministic varying mutation schedules are used to reduce mutation rates, i.e. ${P_{m}}^{\left(0\right)}>{P_{m}}^{\left(T\right)}$. In our case, we also use it to investigate the effect of increasing mutation rate by allowing ${P_{m}}^{\left(0\right)}<{P_{m}}^{\left(T\right)}$.

Table 4 shows the parameter settings Conf1 and Conf2 found by two runs of SMAC using travel time as the observed performance. The settings used by E4DVM are also included for comparison. The settings found by SMAC are different to those used in E4DVM, however, convergence behavior of the algorithm with these configurations and E4DVM are very similar [45]. To better interpret the values of these parameters, Table 5 shows the expected number of mutated signals per solution at time t = 0 and t = T by the two mutation operators in the configurations suggested by SMAC and E4DVM.

**Table 4:** SMAC solutions: EA parameters.

	*P*_*c*_	$P_{m}^{\left(0\right)}$	$P_{m}^{\left(T\right)}$	*P*_*CtO*_	tt avg.
Conf1	0.60	0.93	0.63	0.19	564.10
Conf2	0.50	0.28	0.61	0.29	554.52
E4DVM	1.00	0.29	0.06	0.3	555.90

**Table 5:** Expected number of mutated signals by SMAC configuration and E4DVM.

	NCtOPM		GtM	
	t = 0	t = T	t = 0	t = T
Conf1	12.4	8.4	52.7	35.7
Conf2	5.7	12.4	13.9	30.3
E4DVM	6.1	1.3	14.2	2.9

From Tables 4 and 5 note that the three strategies Conf1, Conf2 and E4DVM mutate green time more often than cycle time, as can be seen by the higher expected numbers of green time mutations compared to the expected numbers of cycle time mutations. Conf2 is a strategy opposite to Conf1 and E4DVM, in the sense that Conf2 increases the expected number of mutations with time whereas Conf1 and E4DVM decrease them. Note that although Conf1 reduces mutations with time similar to E4DVM, the expected number of mutations is significantly larger in the former. See that in Conf1 at t = 0, and t = T the number of expected cycle mutations are two and seven times more than E4DVM, respectively. Similarly, the expected number of green time mutations at t = 0 and t = T are three and twelve times more in Conf1 than in E4DVM.

Though Conf1 and Conf2 mutate more than E4DVM, these strategies apply crossover with rate 0.6 and 0.5, respectively. This means that in the configurations found by SMAC, 40 and 50 percent of the offspring are created applying mutation alone (no crossover), whereas in E4DVM the crossover rate is 1.0 and therefore crossover always precedes mutation. It has been shown that the likelihood of interferences between operators increases when mutation with a high rate per variable follows crossover [33]. This is because a good recombination could be compromised by deleterious mutations or an inefficient recombination could hurt the propagation of beneficial mutations. Thus, smaller rates of crossover combined with higher varying mutations as suggested by SMAC is in accordance with parameter settings that reduce interference between operators. As mentioned above, these three strategies produce similar convergence behavior and lead to a similar low travel time. A more detailed discussion about parameters settings can be found in [45].

### Vehicle movement distribution

In section Initial Mobility Plans for MATSim, Fig 9 shows the overall vehicle movement distribution over time classifying by departures, arrivals and cars en route. In this section, we focus on the peak hour from 07:30 AM to 08:30 AM to illustrate the vehicle movement distribution in the whole network, in a corridor where 15 signals are located, and with more detail in 3 consecutive signals. The data corresponds to an optimized solution of experiment E4DVM with cycle length of 60 seconds (c60E4DVM).

**Fig 16:**
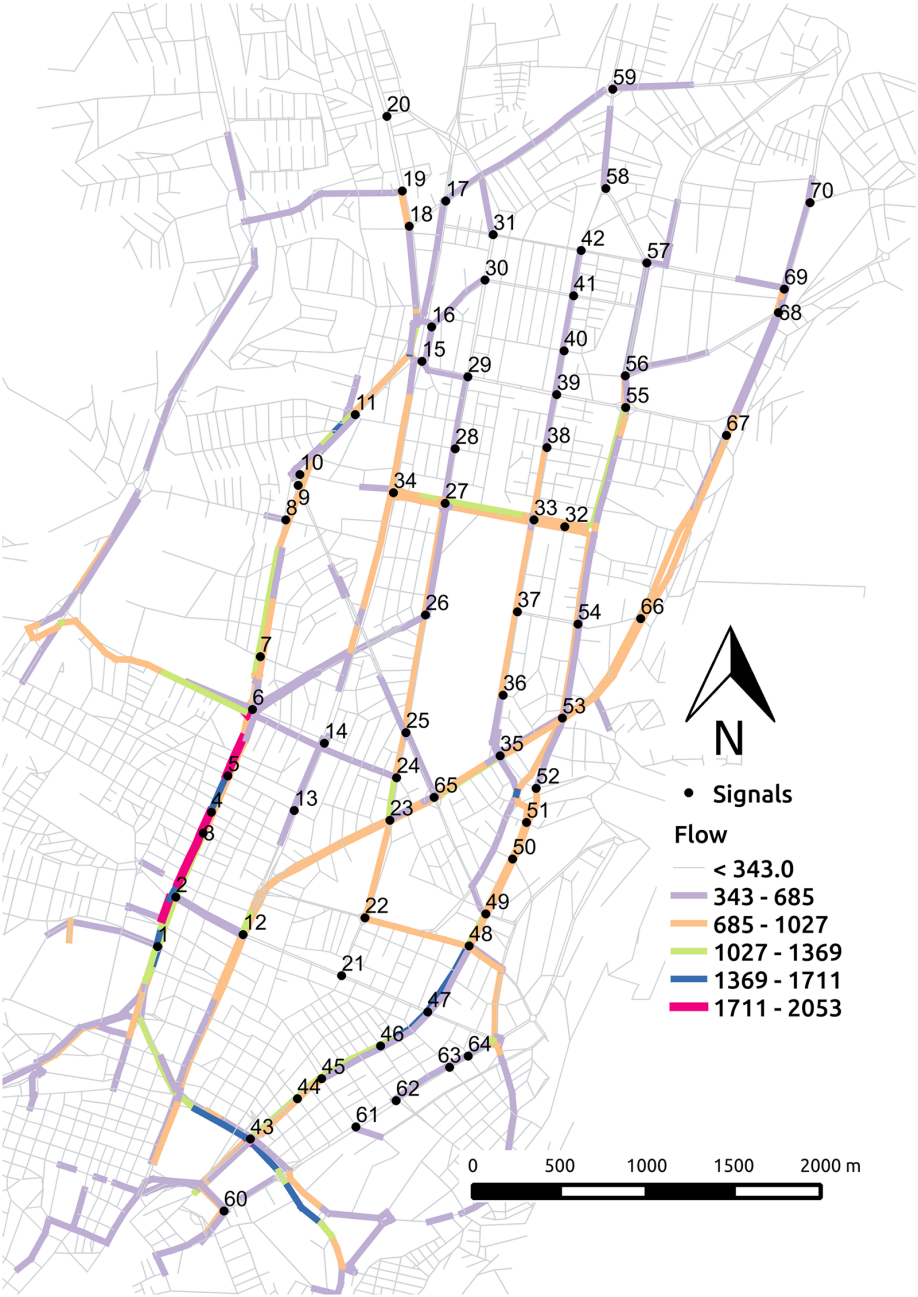
DMQ scenario flow from 07:30AM to 08:30AM.

**Fig 17:**
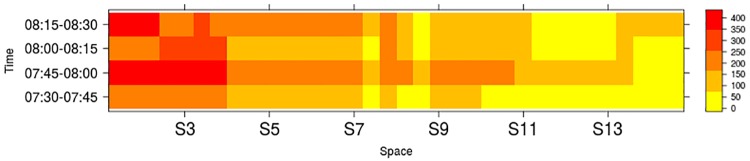
Signals corridor S1-S15.

Fig 16 shows the number of cars per link in the whole network. Links are colored according to the number of cars as shown in the pallete. The numbers close to the black dots identify signalized intersection. Note that in general a larger flow can be observed in the central region than in the perimeter. Also, note that at this hour the largest flow can be seen in the corridor where signals S1 to S6 are located. Fig 17 focuses on the links that belong to the corridor where signals S1 to S15 are located. Here, rows correspond to 15 minutes interval, columns specify links, and colors indicate the number of cars as shown in the pallete.

**Fig 18:**
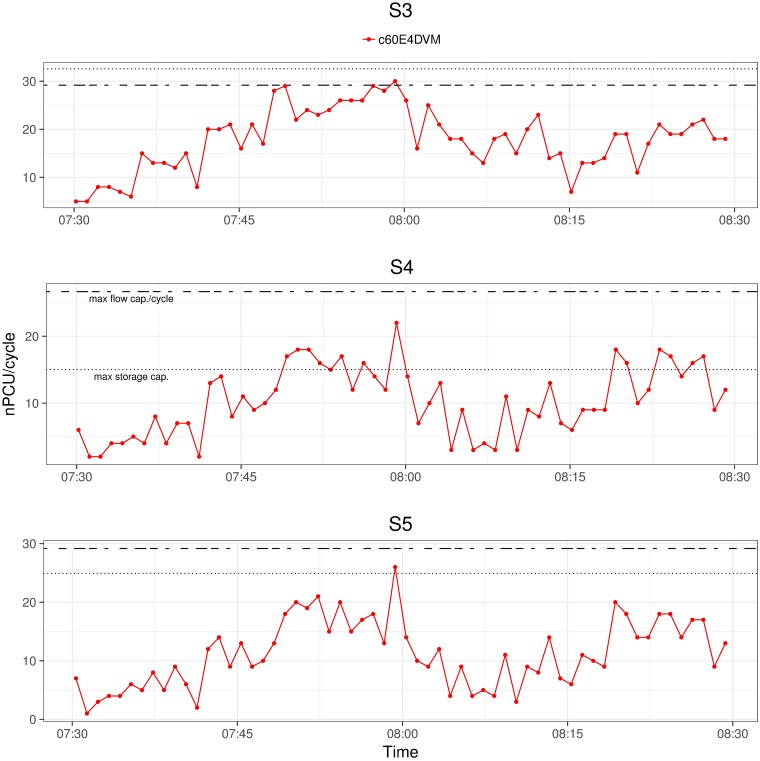
Signals corridor S1-S3 flow from 07:30AM to 08:30AM.

Similarly, Fig 18 shows the number of cars (nPCU) that crossed a signalized intersection over the time at which green time starts. Results are shown for consecutive signalized intersections S3, S4 and S5 with south-north flow. These intersections are located in a zone close to saturation as shown in Fig 16. The storage capacity and max flow capacity per green time of the link where the signal is located are shown as reference in horizontal dotted and dashed lines, respectively. Note that the number of cars counted when they leave the signalized intersections increases from 7:30 to 7:45 and approaches the max flow capacity per green time around 7:50 to 8:00, which is in accordance with Fig 7 in the manuscript.

## Analysis of solutions

In this section, we analyze optimal signal settings found by the algorithm.

### Decision space and cluster analysis

**Fig 19:**
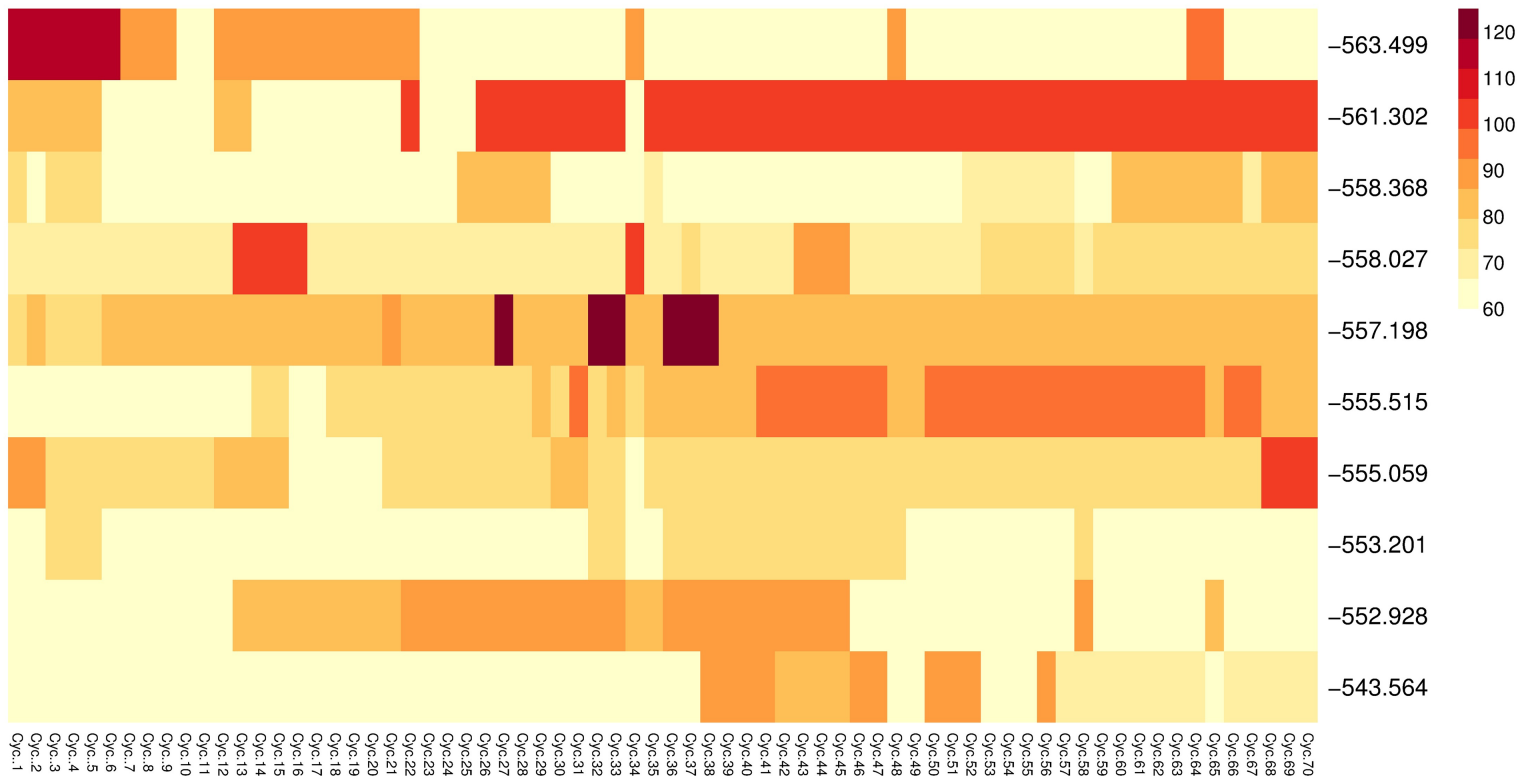
Cycle length of the best solutions for experiment E4DVM scenario S2M. Annotated heatmap: solutions by row (associated travel time in the row label), variables by column (one per signal), values by color (see color legend).

Fig 19 shows the cycle length of the best solutions at the last generation for all runs. Results are shown for experiment E4DVM on the most saturated scenario, S2M. A row corresponds to the settings of all signals in a particular solution and column identifies a signal. The value of cycle length is shown with color as indicated by the color legend. The labels for each row report the travel time of the corresponding solution.

**Fig 20:**
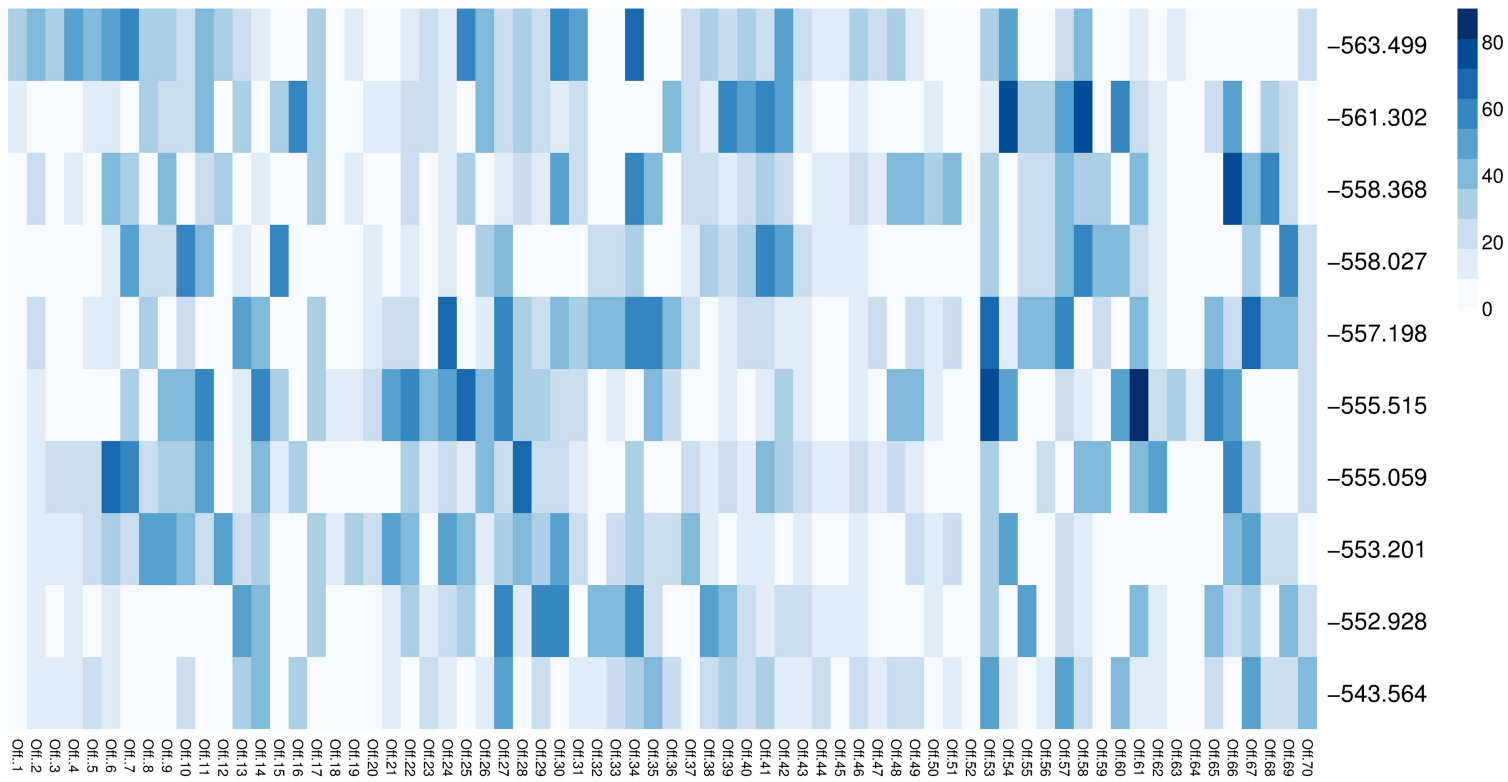
Offset times of the best solutions for experiment E4DVM scenario S2M.

From this figure, note that groups of signals with the same cycle can be seen in the best solutions found by the algorithm in all runs. This is because the *NCtOPM* operator propagates the cycle length value of the reference signal to its neighborhood. However, not all signals have the same cycle. This is an important difference with several approaches that assume one common cycle for all signals [8] based on simple rules for traffic engineering applicable when the number of signals are few and are closely located [47]. Fig 20 shows the values of offsets of the best solutions at the last generation for all runs, similar to Fig 19. Note that there is variability of offset values in each solution due to the operator that adjusts the offset based on the distance between neighbor and the reference signal.

An objective of the neighborhood mutation operator *NCtOPM* was to induce coordination between signals. Figs 19 and 20 suggest that there are some patterns in the settings of cycle length and offsets.

**Fig 21:**
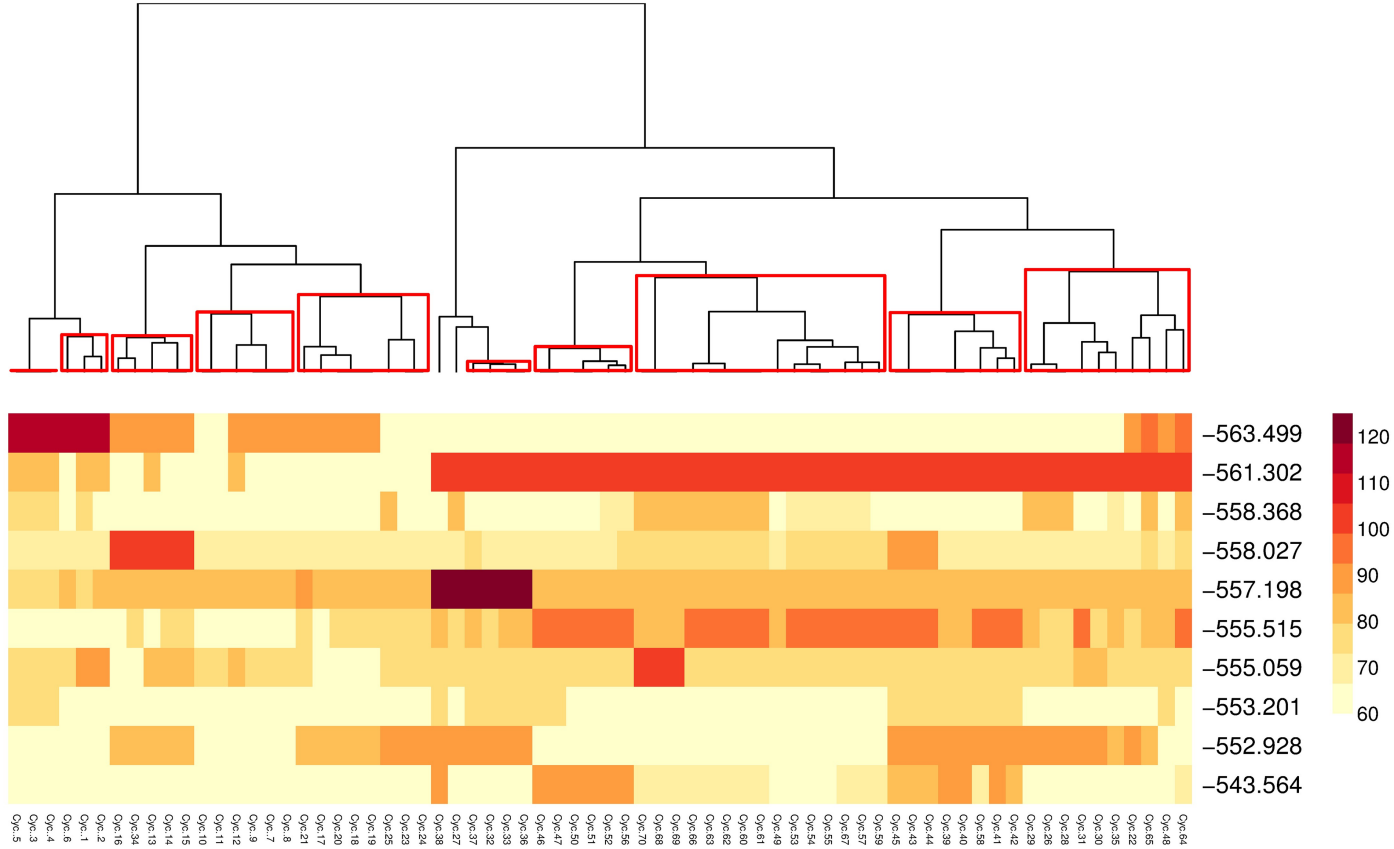
Hierarchical clustering of cycle length of the best solutions for experiment E4DVM scenario S2M.

To distinguish these patterns and extract some design knowledge from them, we cluster signals based on their similarity. Namely, we apply Ward’s agglomerative procedure [48, 49] to create a hierarchy of clusters, using the Euclidean distance between cycle length (or offset) as a measure of similarity. Fig 21 shows the hierarchical clustering of cycle lengths represented as an inverse tree diagram, where the dissimilarity between clusters is proportional to the height at which the branches split. That is, the higher the similarity between signals the lower the split. Below the inverse tree diagram, we show the solutions with the columns reordered according to the clustering.

**Fig 22:**
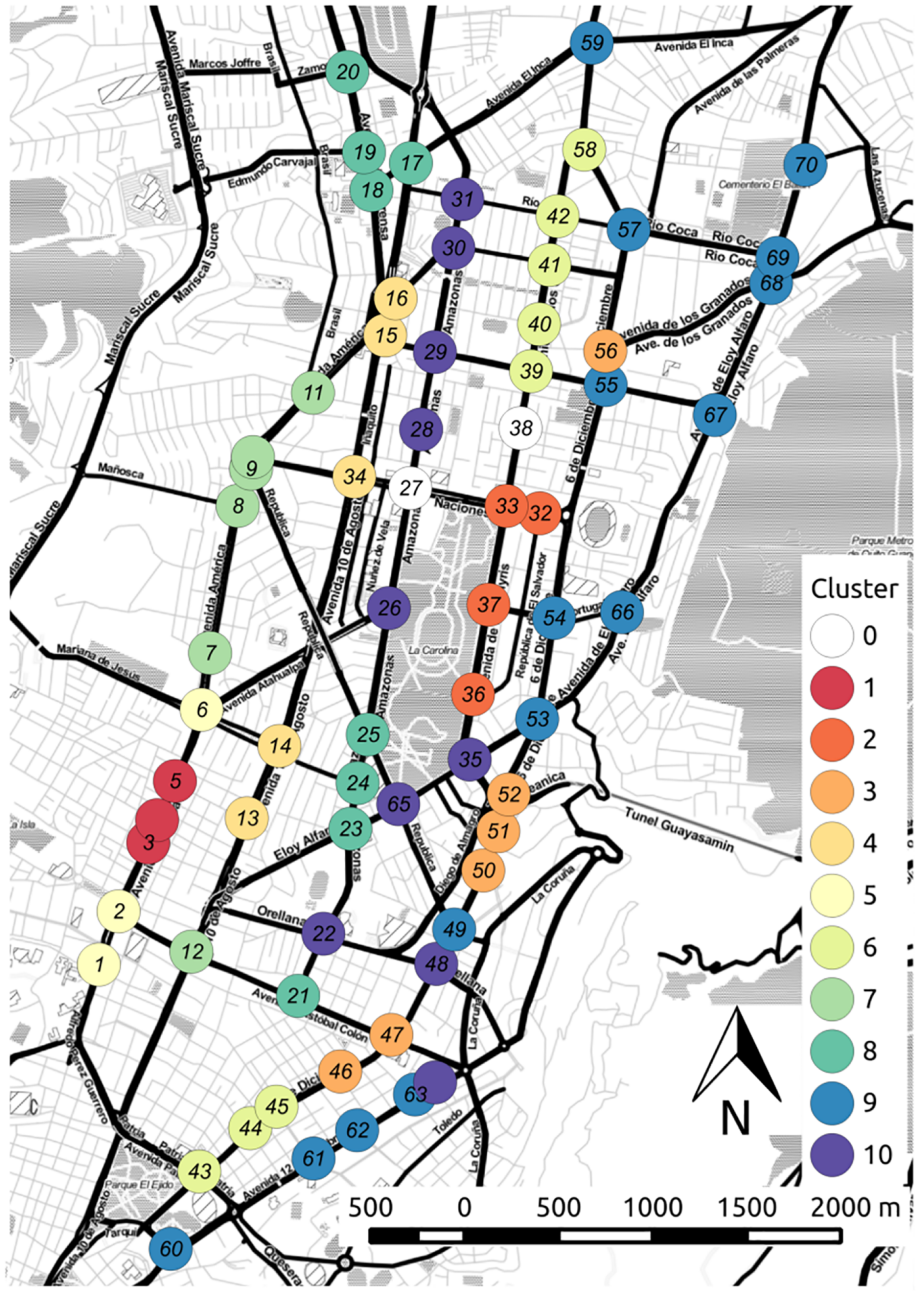
Geolocation of signal clusters, experiment E4DVM scenario S2M.

**Fig 23:**
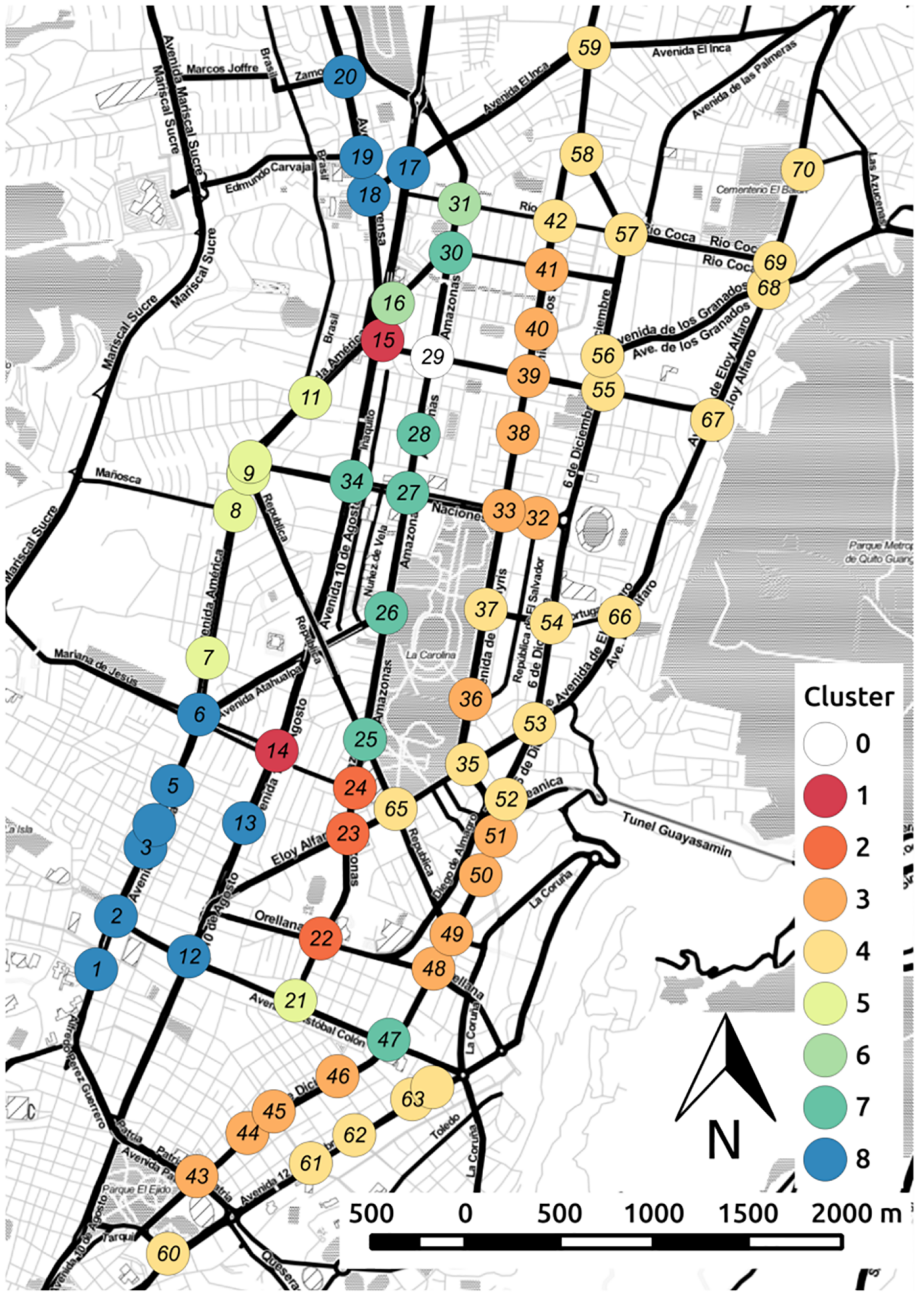
Geolocation of signal clusters, experiment E4DVM scenario S124h.

Bootstrapping is used to test the stability of the assignment of variables to clusters. This also gives us a statistically based criterion to determine where to cut the inverted tree and which clusters to keep [50]. Clusters that are strongly supported by the data are shown in red in the tree diagram. Signals belonging to the selected clusters are coded by color and overlaid on the urban map, as shown in Fig 22. We can see that most clusters of the signals are aligned with the axis SNS of the principal roads of the transport network. Also, large chunks of the clusters are spatially contiguous. Thus, the cluster geolocalization identifies micro-zones with similar characteristics in the city, which can be further analyzed in order to incorporate additional mobility criteria such as safety, emissions and fuel consumption, multi-modality, etc. Similarly, Fig 23 shows the geolocalization of the selected clusters of signals obtained from the best solution of the less saturated experiments E4DVM on S124h. Note that for scenarios under saturated conditions (Fig 22) the number of clusters is higher.

### Emissions and fuel consumption

In this section, we analyze the effects of optimizing traffic light settings on fuel consumption and gas emissions. We use MATSim emission extension [51] that computes the gas emissions per link per agent, at the time an agent enters a link of the transport network. It calculates warm and cold-start exhaust emissions for cars by connecting MATSim simulation output to the detailed database Handbook on Emission Factors for Road Transport (HBEFA) [52]. We have selected seven categories of vehicles based on model year, fuel type and weight. Table 6 shows the distribution of vehicle categories chosen for the scenarios, which is in accordance with census transportation data [38] for Quito city. We assign a category of vehicle to each agent randomly following this distribution.

**Table 6:** Car distribution (fuel = gasoline and weight ≦ 2Tons).

Year-Category	%	Year-Category	%
2000-2002—Euro2	24.40	2008-2010—Euro5	13.70
2002-2004—Euro3	7.80	2010-2012—Euro6	23.80
2004-2006—Euro4	12.20	2012-2014—Euro6	5.20
2006-2008—Euro4	13.00		

MATSim computes warm emissions deriving the kinematic characteristics from the simulation and combines this information with vehicle characteristics to extract emission factors from the database of the Handbook on Emission Factors for Road Transport (HBEFA). To derive the kinematic characteristics, the emission model considers ‘free flow’ and ‘stop&go’ as traffic states per road segment. To calculate cold-start emissions, MATSim derives parking duration and accumulated traveled distance from the simulation. For parking duration, HBEFA database differentiates emission factors in one hour time steps from 1h to 12h. After 12 hours the vehicle is fully cooled down. There are also different cold emission factors for short trips (less than 1Km) and longer trips (greater than 1Km) [53].

Table 7 shows travel time (TT) and fuel consumption (FC) together with HC, CO, NOx, and *CO*_2_ emissions produced by all agents on S124h. First, we show as reference results corresponding to the equilibrium state without traffic signals and the solution with smallest travel time at generation 0 when traffic signals are included. Note that at generation 0 all signals are set with the same cycle length, as explained before. Next, we show results for solutions with traffic signals that minimize travel time at generation 50 in experiments E4 and E4DVM. Comparing with generation 0, results at generation 50 illustrate that in addition to minimizing travel time, fuel consumption and the various kinds of emissions can also be reduced significantly if traffic lights are optimized. Further, the experiments E4 and E4DVM represents an incrementally better optimization process for TT, as discussed above. Note that FC and emissions also reduce incrementally in these experiments. Thus, a better optimization process for TT, given by the neighborhood operators and deterministic varying mutation, is also highly correlated to a better optimization for FC and emissions.

**Table 7:** Emissions of best solutions on scenario S124h.

		g = 0	g = 50	
	Eq.St.	C_h_ = 130	E4	E4DVM
TT	591.28	1302.06	694.17	700.19
FC	14275.90	15201.26	14961.03	**14864.17**
CO2	26960.42	29150.52	28434.11	**28348.46**
PM	0.28	0.29	0.29	0.29
NOx	32.41	33.38	33.06	**33.01**
NO2	1.08	1.14	1.12	1.12
SO2	0.13	0.14	0.14	0.14
CO	772.98	774.13	773.96	**773.91**

FC in Liters—Emission in Kg.—TT in s.

Similarly, Table 8 shows results for experiments E4 and E4DVM with one point (XP1) and two point crossover (XP2) on the most saturated scenario S2M. Note from this table that significant reductions in FC and emissions can also be obtained in this kind of saturated scenarios.

**Table 8:** Emissions of best solutions on scenario S2M.

		g = 0		g = 50	
	Eq.St.	C_h_ = 85	E4	E4DVM(XP1)	E4DVM(XP2)
TT	386.63	650.62	530.96	517.98	524.11
FC	6356.91	6883.16	6771.98	**6746.11**	**6746.52**
CO2	14903.61	16553.44	16204.88	**16123.79**	**16125.08**
PM	0.15	0.16	0.16	0.16	0.16
NOx	15.75	16.55	16.40	16.36	16.36
NO2	0.55	0.59	0.58	0.58	0.58
SO2	0.07	0.08	0.08	0.08	0.08
CO	482.93	484.14	484.09	484.01	483.99

FC in Liters—Emission in Kg.—TT in s.

### Spatial analysis for CO_2_ emissions

In the following, we analyze carbon dioxide emissions (*CO*_2_) on a spatially disaggregated level for scenario S2M. MATSim output emissions are aggregated per link for all agents. For geolocation on the urban area of the scenario, *CO*_2_ emissions are spatially smoothed using the inverse distance weighted interpolation method [54]. For that, we used as sampling points the centroid of each link and its corresponding accumulated measure of emissions.

Our spacial analysis of the emissions is not an indication of how the emissions are being dispersed in the atmosphere, instead, we illustrate the origin and intensity of the emissions to compare an optimized traffic signal plan against a not optimized one.

**Fig 24:**
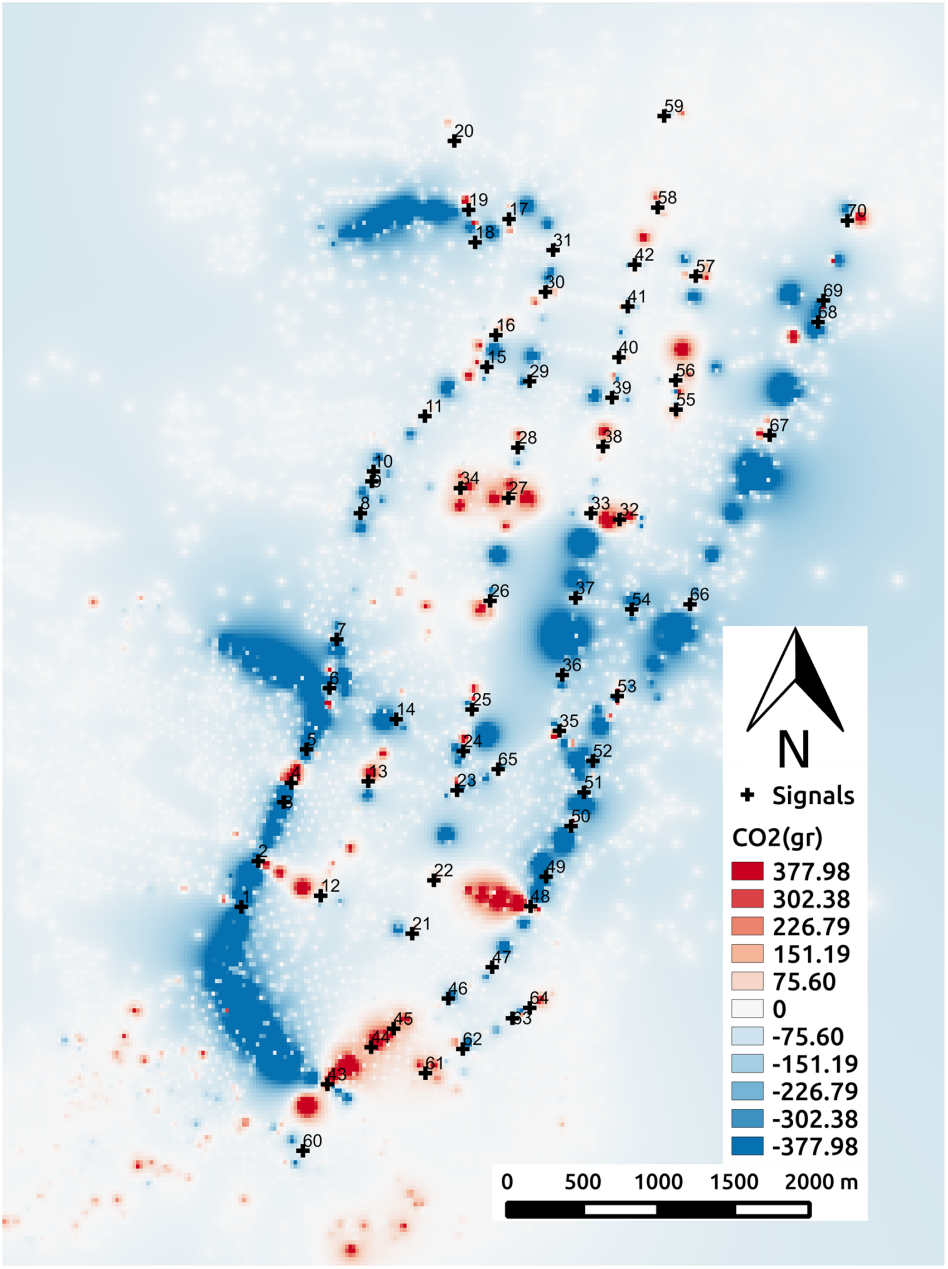
Change in CO_2_ emissions.

Fig 24 shows the difference in *CO*_2_ emissions between the best solution in the initial population, where all signals have the same cycle *C*_*h*_ = 85, and the best solution at the last generation of experiment E4DVM. We use the single-value cycle length (*C*_*h*_ = 85) as reference for comparison to show the improvement that can be achieved from same-cycle solutions at the initial population and to highlight the relevance of having different cycle lengths for different clusters of signals at the end of evolution.

We use a red-blue palette to show emission differences, where levels of red represent increments and levels of blue represent reductions of emissions. White is used to show no difference in emissions between the two cases. Note the dark blue regions, which show that emissions mostly reduce across the area of study when signals settings are optimized. However, note that in some regions there is a relative increase in emissions. This figure gives an overall view of the effects of optimization in *CO*_2_ emissions. It provides useful information for city planners and can be used to feedback the evolutionary algorithm to favor low emissions in certain regions of interest.

### Coordination analysis

An important aspect of signal coordination is improving the traffic flows and reduce congestion. A continuous traffic flow during several intersections in one main direction is usually referred as a *green wave*. In this section, we analyze the optimized signal settings to determine if their coordination induce green waves or not.

**Fig 25:**
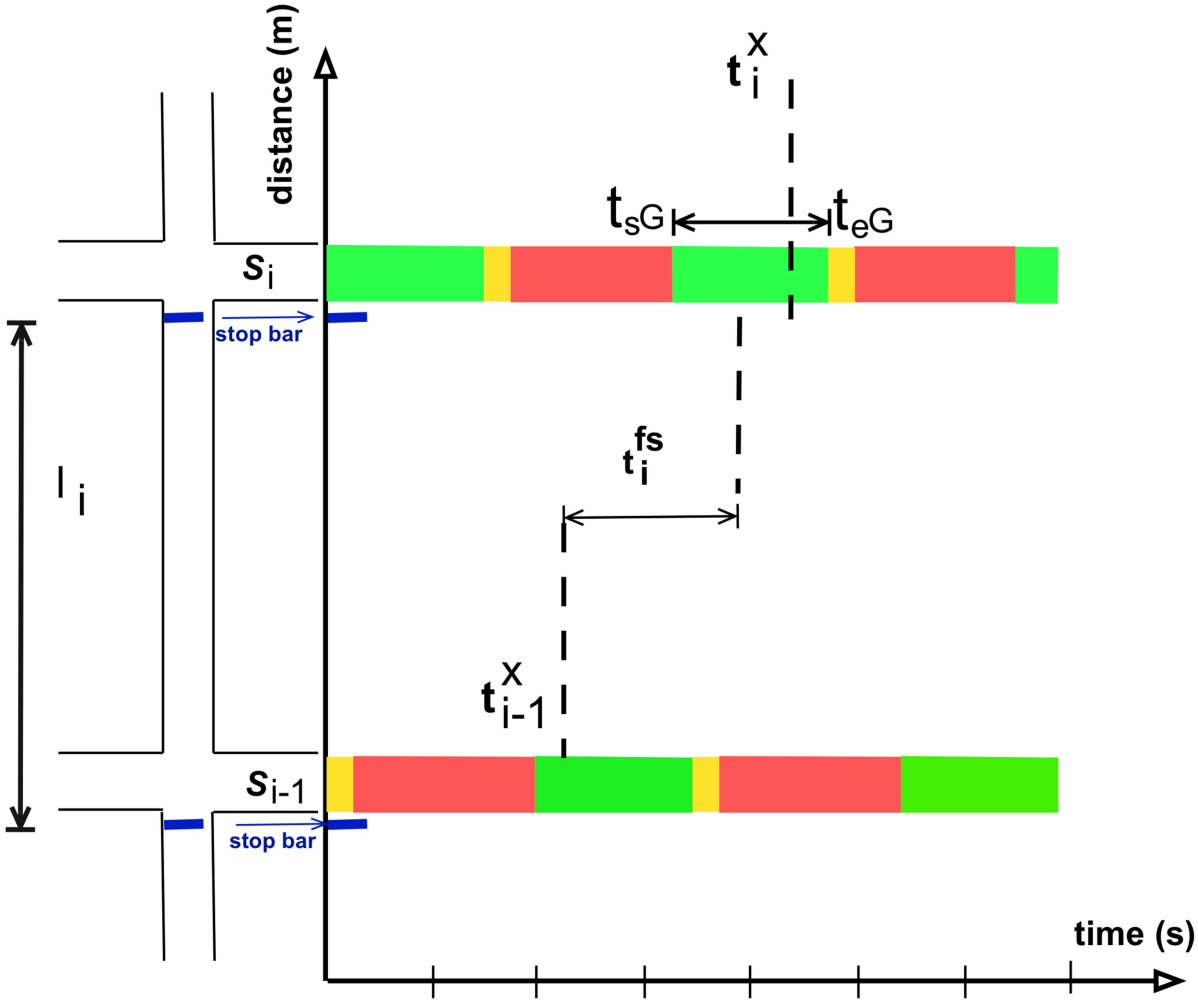
Variables to detect green phase crossing.

Let us denote *l*_*i*−1_ and *l*_*i*_ two links with consecutive signals *S*_*i*−1_ and *S*_*i*_ located at the end of each one of them, as illustrated in Fig 25. The time at which an agent exits link *l*_*i*_ and crosses signal *S*_*i*_ is $t_{i}^{x}$. The starting and ending time of the green time phase when the agent crosses signal *S*_*i*_ are *t*_*sG*_ and *t*_*eG*_, respectively.

The earlier expected arrival time to signal *S*_*i*_ after crossing signal *S*_*i*−1_ is $t_{i-1}^{x}+t_{i}^{fs}$, where $t_{i}^{fs}$ is the time that takes to cover the distance of link *l*_*i*_ traveling at free speed, i.e. the maximum speed at which an agent can circulate at a given link.

In this work, an agent is said to cross two consecutive signalized intersections in an uninterrupted flow if the earlier expected arrival time to signal *S*_*i*_ and the actual exit time from link *l*_*i*_ (cross signal *S*_*i*_) are within the green phase window of signal *S*_*i*_, i.e.

$$t_{sG}\leq t_{i-1}^{x}+t_{i}^{fs}\leq t_{eG}\wedge t_{sG}\leq t_{i}^{x}\leq t_{eG}$$ (12)

**Fig 26:**
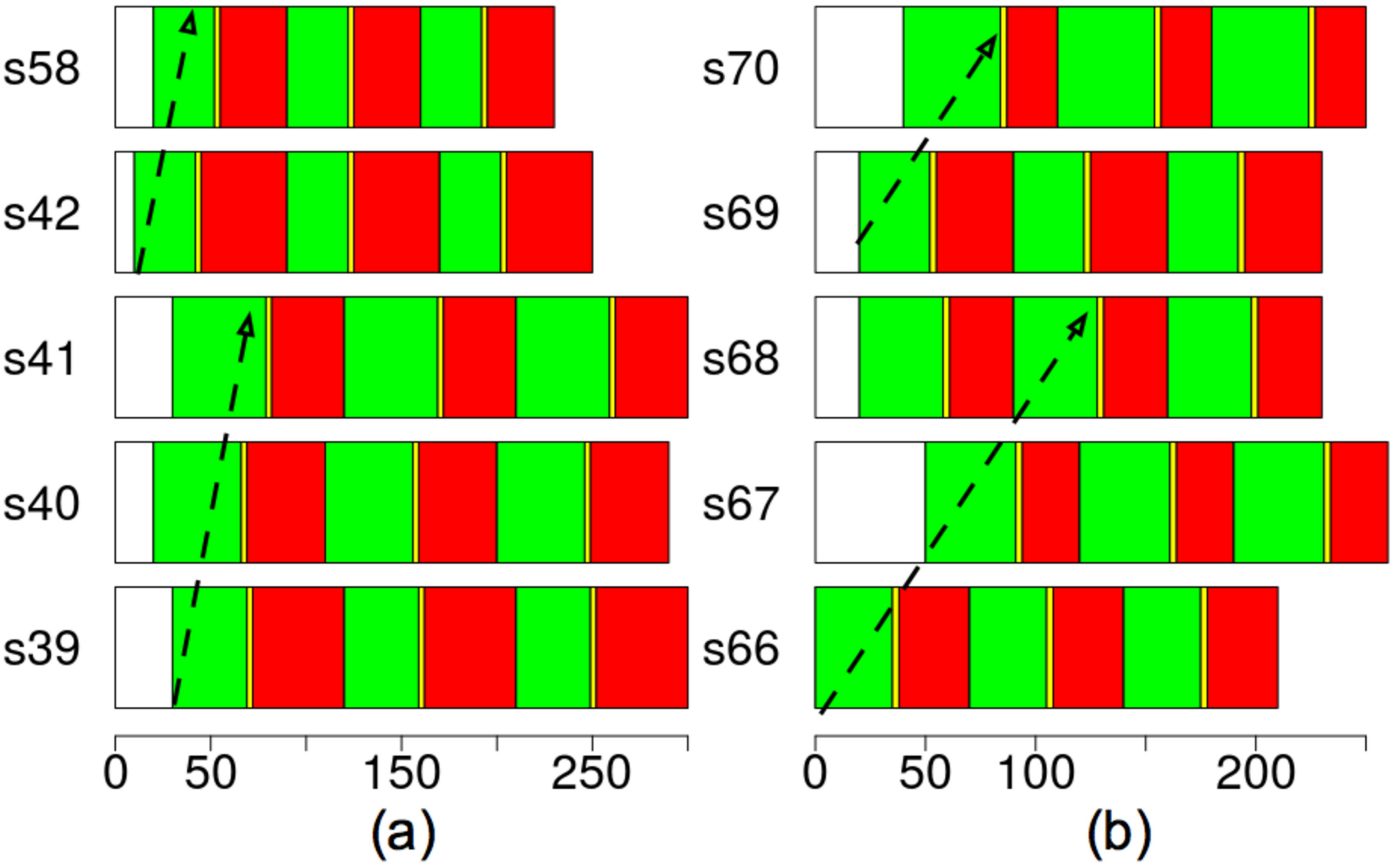
Best individual (lowest travel time) cluster No.6 (a) and cluster No. 9 (b) for south-north direction, experiment E4DVM, scenario S2M.

Fig 26 represent the phenotype expression of some consecutive signals of the best individual of experiment E4DVM on the saturated scenario S2M. Fig 26(a) shows in different rows the offset and three cycles (green, inter-green, red) of consecutive geolocated signalized intersections s39–s42 and s58 that belong to cluster 6 as shown in Fig 22. Similarly, Fig 26(b) shows consecutive geolocated signalized intersections s66–s70 that belong to cluster 9. A visual inspection of these signals settings suggests that green waves could emerge from them. To verify this, we count the number of agents that cross these signals.

Tables 9 and 10 show the percentage of agents that cross two or more consecutive green signals. In addition, it shows in detail the number of agents that cross 2, 3, 4 and 5 consecutive signals. Results are shown for the best solution in the initial population, where cycle length of all signals is *C*_*h*_ = 85, and the best solutions of experiments E4 and E4DVM. Note that in cluster 6 there is not much difference in percentage between the initial solution and the optimized ones, although for E4DVM some agents can cross up to 4 consecutive signals. On the other hand in cluster 9 there is a significant increase in percentage in the optimized solutions and some agents can cross up to 4 or 5 consecutive signals. Looking at Fig 24 note that in the region where cluster 6 is located there is no reduction in *CO*_2_ emissions. However in the area where cluster 9 is located there is a significant reduction in emissions.

**Table 9:** Green waves cluster 6, total agents = 253.

gw	C_h_85	E4	E4DVM
%	0.49	0.42	0.47
2	137	123	73
3	20	–	43
4	–	–	14

**Table 10:** Green waves cluster 9, total agents = 1400.

gw	C_h_85	E4	E4DVM
%	0.39	0.45	0.68
2	554	377	556
3	60	230	245
4	–	18	127
5	–	1	61

## Conclusions and future work

This work presented a design optimization framework for the transportation system of Quito. An evolutionary algorithm to search efficiently using small populations in few generations was proposed. The algorithm was coupled to the multi-agent transport simulator MATSim to study the optimization of a large number of traffic signal controls located on a wide area of the city. Three mobility scenarios of 20,000 agents modeled based on activities were used to verify the effectiveness of the evolutionary algorithm. It was shown that green times should be mutated with higher probability than cycle and offsets, the propagation of cycle length to neighboring signals with offsets set based on distance leads to better coordination of signals, and the use of varying mutation increases convergence speed. The combination of these strategies efficiently and effectively explore the large search space of traffic signal parameters. The effect of crossover was also verified, showing that better convergence can be achieved when the crossover is activated, although no significant difference between one and two point crossover was observed. The proposed algorithm combines a strong selection pressure given by elitism with crossover followed by varying mutation, where an appropriate balance between exploration and exploitation can be achieve in several ways. An analysis of the parameters of the algorithm using sequential model based algorithm configuration (SMAC) confirmed our finding that mutation of green times should be emphasized over cycle length and offset propagation. It also showed that other configurations with varying mutations relatively higher than the suggested in our configuration but using smaller rates of crossover lead to similarly good results.

Also, hierarchical clustering was performed on the best solutions found in several runs of the algorithm. An analysis of signal clusters and their geolocation, estimation of fuel consumption, spatial analysis of emissions, and an analysis of signal coordination provided an overall picture of the systemic effects of the optimization process. It also showed that the proposed approach is helpful to deepen our understanding of the problem and gain knowledge about the system.

There is ongoing work on multi-objective formulations of the traffic signals problem, where criteria for sustainable transport systems are considered as objectives to be optimized.

In the future, we would like to include the multi-modality of the transport network and study ways to improve the sustainability of Quito’s transport and mobility system. Additionally, we would like to include signals with more than two phases. Also, it will be important to combine adaptive signals with default pre-fixed timing plans that are optimal under certain conditions or scenarios.

## Supporting information

S1 File DMQ scenario data sets. (GZ)
